# Generation of functional cardiomyocytes from rat embryonic and induced pluripotent stem cells using feeder-free expansion and differentiation in suspension culture

**DOI:** 10.1371/journal.pone.0192652

**Published:** 2018-03-07

**Authors:** Julia Dahlmann, George Awad, Carsten Dolny, Sönke Weinert, Karin Richter, Klaus-Dieter Fischer, Thomas Munsch, Volkmar Leßmann, Marianne Volleth, Martin Zenker, Yaoyao Chen, Claudia Merkl, Angelika Schnieke, Hassina Baraki, Ingo Kutschka, George Kensah

**Affiliations:** 1 Clinic of Cardiothoracic Surgery, Otto-von-Guericke University Magdeburg, Magdeburg, Germany; 2 Clinic of Cardiology and Angiology, Otto-von-Guericke University Magdeburg, Magdeburg, Germany; 3 Institute of Biochemistry and Cell Biology, Otto-von-Guericke University Magdeburg, Magdeburg, Germany; 4 Institute of Physiology, Otto-von-Guericke University Magdeburg, Magdeburg, Germany; 5 Institute of Human Genetics, Otto-von-Guericke University Magdeburg, Magdeburg, Germany; 6 Wellcome Trust-Medical Research Council Stem Cell Institute, University of Cambridge, Cambridge, United Kingdom; 7 Chair of Livestock Biotechnology, Technical University Munich, Freising-Weihenstephan, Germany; University of Texas at Austin Dell Medical School, UNITED STATES

## Abstract

The possibility to generate cardiomyocytes from pluripotent stem cells *in vitro* has enormous significance for basic research, disease modeling, drug development and heart repair. The concept of heart muscle reconstruction has been studied and optimized in the rat model using rat primary cardiovascular cells or xenogeneic pluripotent stem cell derived-cardiomyocytes for years. However, the lack of rat pluripotent stem cells (rPSCs) and their cardiovascular derivatives prevented the establishment of an authentic clinically relevant syngeneic or allogeneic rat heart regeneration model. In this study, we comparatively explored the potential of recently available rat embryonic stem cells (rESCs) and induced pluripotent stem cells (riPSCs) as a source for cardiomyocytes (CMs). We developed feeder cell-free culture conditions facilitating the expansion of undifferentiated rPSCs and initiated cardiac differentiation by embryoid body (EB)-formation in agarose microwell arrays, which substituted the robust but labor-intensive hanging drop (HD) method. Ascorbic acid was identified as an efficient enhancer of cardiac differentiation in both rPSC types by significantly increasing the number of beating EBs (3.6 ± 1.6-fold for rESCs and 17.6 ± 3.2-fold for riPSCs). These optimizations resulted in a differentiation efficiency of up to 20% cTnT^pos^ rPSC-derived CMs. CMs showed spontaneous contractions, expressed cardiac markers and had typical morphological features. Electrophysiology of riPSC-CMs revealed different cardiac subtypes and physiological responses to cardio-active drugs. In conclusion, we describe rPSCs as a robust source of CMs, which is a prerequisite for detailed preclinical studies of myocardial reconstruction in a physiologically and immunologically relevant small animal model.

## Introduction

Laboratory rats are a fundamental tool in the investigation of heart physiology, heart failure and myocardial injury with profound advantages over mice. Open-chest cardiac surgery and invasive hemodynamic assessment are easier to pursue in this larger rodent model [1,2]. Rat models have been used extensively for proof-of-principle cell and tissue based regenerative studies of the heart [3–6] and continue to be highly attractive, especially since the induced pluripotent stem cell (iPSC) technology has driven the focus even more towards the strategy of stem cell-mediated heart repair [7–11]. A significant limitation is the fact that published studies invariably represent xenogeneic models, e.g. using either human or murine iPSC-derived cardiomyocytes for transplantation. In these settings, significance is impaired by species differences in immunology, genetics and cardiac physiology. It is only recently that germline competent rat embryonic stem cells (rESCs) [12,13], and induced pluripotent stem cells (riPSCs) [14,15] are available. In combination with novel possibilities of generating transgenic rat strains via novel genome editing strategies [16], this will increase opportunities for the refinement of treatment strategies of various degenerative diseases. However, until now, data on the cardiac differentiation potential of rat pluripotent stem cells are limited, with only one study describing the generation of functional cardiomyocytes from rESCs [17]. The potential of riPSCs to generate cardiomyocytes (CMs) remains elusive. One possible reason for this lack of knowledge is that conditions for culturing and genetic modification of undifferentiated rPSCs had to be optimized [18,19]. Additionally, formation of stable embryoid bodies (EBs) of undifferentiated rPSCs to induce differentiation was a general problem reported by several groups [12,19,20]. In contrast to murine and human PSCs, where defined protocols meanwhile result in high cardiac differentiation efficiencies with e.g. 40–60% CMs for mESCs [21,22] and >80% for hESCs and hiPSCs [23,24], the reported efficiency for rESCs is relatively low. Here, an average of 6.6% cardiac Troponin T (cTnT)^pos^ CMs has been observed in the differentiated population [17]. This is hardly enough to provide sufficient numbers of PSC-derived CMs for allogeneic or syngeneic transplantation studies. We assume that, depending on the delivery method, at least 2-10x10^6^ PSC-CMs per animal will be required to achieve relevant restoration of infarcted myocardium [7,25].

Therefore, in this study we aimed at establishing a robust *in vitro* protocol for expansion and cardiac differentiation of rPSCs. In a side-by-side comparison of rESCs and riPSCs, we assessed the effects of a simplified expansion method on rPSC stability. Furthermore, we sought to optimize cardiac differentiation efficiencies to provide sufficient numbers of rPSC-CMs for future myocardial reconstruction approaches in a clinically relevant syngeneic or allogeneic model. Additionally, morphology and physiology of iPSC-CMs was analyzed in detail to assess functionality.

## Materials and methods

### Expansion of undifferentiated rESCs and riPSCs in feeder-dependent and feeder-free culture

rESCs were derived from blastocysts of female Dark Agouti rats as described previously [26]. The male riPSC clone T1/64 was reprogrammed from rat adipose tissue-derived mesenchymal stem cells (rADMSCs) from Fischer 344 rats using a non-viral vector containing the murine reprogramming factors Oct4, Sox2, c-Myc and Klf4 controlled by a bidirectional doxycycline-inducible promoter, as described elsewhere [20]. For feeder-dependent and feeder-free culture, N2B27/LIF2i expansion medium was composed of a 1:1 mixture of N2-Medium (DMEM/F12, 1x N2 supplement, 100 μg/mL BSA fraction V) and B27 medium (Neurobasal medium, 1x B27 supplement w/o retinoic acid, 2 mM Glutamax; all Thermo Scientific) supplemented with 0.1 mM 2-mercaptoethanol, 1000 U/mL LIF (ESGRO, Millipore), 3 μM glycogen synthase kinase 3 (GSK3β) inhibitor CHIR99021 (Axon Medchem) and 0.5 μM mitogen-activated protein kinase kinase (MEK1/2) inhibitor PD0325901 (Sigma). rPSCs were cultured on a high-density feeder layer (7x10^5^ cells/cm^2^) of mitotically inactivated gamma-irradiated (30 Gy) murine embryonic fibroblasts on 0.1% gelatin coated tissue culture plates. Partial medium exchange was conducted daily. Every four days, floating and loosely attached colonies were harvested by gentle washing, dissociated with Accutase (Thermo Scientific) and plated again (at 5x10^3^ cells/cm^2^) onto a fresh feeder layer. For feeder-free monolayer-based expansion of rPSCs, T25 flasks (TPP) were coated for 1 h with a 1:400 dilution (200 μL/cm^2^) of Geltrex dissolved in DMEM/F12 + Glutamax (both Thermo Scientific). rPSCs were seeded at a density of 1.6x10^4^ (rESC) or 1.2x10^4^ (riPSC) cells per cm^2^ and passaged every 3–4 days using Accutase.

### Karyotype analysis

For each karyotyping approach, metaphases of at least 100 undifferentiated rPSCs were examined. Cells were treated with a final concentration of 0.1 μg/mL Colcemid (Invitrogen) for 2 h in N2B27/LIF2i. Cells were detached with trypsin/EDTA (0.05/0.02%, Biochrom). After centrifugation, the pellet was incubated for 15 min at 37°C in hypotonic solution (0.32% KCl with 0.2% (v/v) fetal calf serum (FCS)). Cells were fixed in ice-cold methanol/acetic acid, 3:1. G-banding was performed according to Seabright [27]. For visualizing of metaphase images and karyograms the IKAROS software of MetaSystems (Altlußheim, Germany) was used. Chromosomes were arranged according to Hamta et al. [28].

### Microsatellite genotyping

Genomic DNA was isolated from undifferentiated rPSC and rESCs using the QuickExtract DNA Extraction Solution (Epicentre) and 200 ng DNA was used as template for amplification. Rat strain specific alleles, primer sequences and PCR conditions are listed in S1 Table.

### Cardiac differentiation of rESCs and riPSCs

To determine cardiac differentiation efficiency of rPSCs in small scale, the hanging drop (HD) method was employed using 3x10^3^ undifferentiated rPSCs / droplet in 33 μL of differentiation medium which was composed of IMDM + Glutamax, 1% (v/v) non-essential amino acids, 0.2 mM L-Glutamine, 0.1 mM 2-mercaptoethanol (all Thermo Scientific) and 15% (v/v) of a lot of pretested FCS (S2 Table, Sigma, F7524, Lot BCBQ7890V). Following 2 days in HDs, EBs were transferred individually to 96 well plates (Thermo Scientific) that was coated with 1% (w/v) agarose/IMDM to avoid attachment of EBs. Each 96-Well contained 130 μL differentiation medium, supplemented with 100 μM L-ascorbic acid-2-phosphate sesquimagnesium salt hydrate (AA-2P, Sigma). The emergence of beating EBs was assessed by microscopic evaluation every second day from day 8 to day 14.

For large-scale cardiac differentiation, we used the agarose microwell method that we previously developed for EB formation of mouse and human PSCs [29,30]. In brief, 1.5% (w/v) agarose (NEEO ultra quality, Roth) was dissolved by boiling in IMDM + Glutamax. The liquid agarose/IMDM solution was filter-sterilized and cooled to 65°C. Two mL of this solution were applied to a 12-Well plate (Nunc) and the structured surface of a sterilized silicon master (generated from Aggrewell800 plates (Stem Cell Technologies) by soft lithography) was carefully placed into this solution. After solidification of the agarose, the silicone master was removed resulting in agarose microwells containing approximately 300 micro-cavities. Agarose microwells were equilibrated for 2 h with rat differentiation medium. For the generation of EBs, agarose microwells were seeded with 2 x 10^3^ and 3 x 10^3^ undifferentiated rPSCs. Cells settled by gravitation and formed aggregates, which were harvested after 48 h and transferred to 10 cm Petri dishes with 10 mL differentiation medium and 100 μM AA-2P. Subsequent culture was conducted in dynamic suspension culture on an orbital shaker (Infors, Celltron) at 60 rpm. The percentage of beating EBs was assessed on day 14–16 by microscopic evaluation.

### Alkaline phosphatase detection and immunofluorescence staining

Alkaline phosphatase staining was performed using the Alkaline phosphatase detection kit (EMD Millipore) according to the manufacturer’s instructions. For immunofluorescence staining, cells and cryosections (10 μm) of OCT-embedded embryoid bodies (Tissue-Tek, Sakura) were fixed for 20 min with 4% (w/v) paraformaldehyde at room temperature and blocked with phosphate buffered saline (PBS) containing 5% (v/v) goat and donkey serum (Millipore), respectively, and 0.25% (v/v) Triton X-100 (Sigma). Cells were incubated for 1 h or overnight with a primary antibody diluted in PBS containing 1% (w/v) bovine serum albumin at room temperature or 4°C, followed by three washing steps with PBS and further incubation (30 min) with the appropriate fluorescent secondary antibody (Jackson Immunoresearch) at room temperature. For antibodies and dilutions see S3 Table). Nuclei were counterstained with 4’, 6’-diamidino-2-phenylindole (DAPI, Sigma-Aldrich) and analyzed using an EVOS FL Auto fluorescence microscope (Life Technologies). Projection areas of EBs were evaluated using ImageJ.

### Flow cytometry

Undifferentiated rPSC colonies and monolayers were dissociated with Accutase (Thermo Scientific). rPSC-derived EBs (day 14) were dissociated after washing in PBS w/o Ca^2+^ and Mg^2+^ for 45 min using 1 mg/mL collagenase B (Roche) in low calcium solution (mM: 120 NaCl, 5.4 KCl, 5 MgSO_4_, 5 Na-pyruvate, 20 glucose, 20 taurine, 0.3 CaCl_2_ and 10 HEPES at pH 6.9) in a thermomixer at 1200 rpm. For immuno-staining, cells were fixed with 2% (w/v) paraformaldehyde for 10 min at room temperature and permeabilized with chilled methanol for 10 min. Primary and secondary antibodies were diluted in PBS. Flow cytometry analysis was performed using a FACSCanto II (Beckton Dickinson) and FlowJo software (version 9.4.10; Tree Star Inc.). Primary and secondary antibodies used are listed in S3 Table.

### Transmission electron microscopy (TEM)

Beating EBs were fixed with 4% (w/v) formaldehyde (FA) in phosphate buffer (pH 7.4) for 10 min at room temperature followed by a mixture of 1.5% (w/v) glutaraldehyde und 2% (w/v) FA, for 2 h at 6°C. After three washing steps in 0.1 M cacodylate buffer, EBs were incubated for 60 min in 1% (w/v) OsO4 and 0.4% (w/v) potassium ferrocyanide in 0.1 M cacodylate buffer, washed and dehydrated in a graded ethanol series including a staining with 2% (w/v) uranyl acetate in 70% ethanol for 40 min. After incubation in propylene oxide, EBs were transferred to Durcupan (Electron microscopy sciences, EMS, USA), infiltrated over night at room temperature and embedded in molds filled with Durcupan. After polymerization at 60°C for 48 h, resin blocks were trimmed and ultrathin sections (~ 70 nm) were prepared with an ultramicrotome (EM UC6; Leica, Germany) and examined with a EM900 transmission electron microscope (Zeiss, Germany). Images were taken with a mounted wide-angle 2k-CCD-dual speed camera (TRS, Germany) using the SPImage Viewer software (TRS/SysProg, Germany/Belarus).

### Analysis of mRNA expression

Total RNA was isolated from undifferentiated rPSCs and EBs from days 3–28 of differentiation using TRIZOL Reagent. DNA was digested by DNAse I for 30 min at 37°C. DNA-free RNA (500 ng) was directly used for first strand cDNA synthesis using random hexamer primers with the RevertAid^™^ First Strand cDNA Synthesis Kit (all reagents: Thermo Scientific). Samples without reverse transcriptase incubation (RT-) served as negative controls to detect contamination by genomic DNA. For polymerase chain reaction, cDNA was amplified using DreamTaq Green PCR Mastermix (Thermo Scientific) and 1 μM of each primer (MWG) in a 25 μL reaction using a PTC-200 Peltier Thermal Cycler (MJ Research). Primer pairs and individual annealing temperatures can be found in S1 Table.

### Whole-cell patch clamp analysis

For measurements of cardiac action potentials, contracting riPSC-derived EBs were dissociated on day 18 of differentiation and plated onto fibronectin coated glass coverslips. Three to four days after plating, coverslips were mounted on the microscope stage (Axioskop, Zeiss), equipped with a temperature-controlled (35°C) recording chamber and perfused continuously with extracellular solution. Patch pipettes were pulled from borosilicate glass (GC150T-10, Clark Electromedical Instruments) to resistances of 3–5 MΩ and filled with (in mM): 50 KCl, 80 K-aspartate, 5 MgCl_2_, 5 EGTA, 10 HEPES, and 5 Na_2_ ATP, adjusted to pH 7.2 with KOH. The extracellular bathing solution contained (in mM): 135 NaCl, 5.4 KCl, 1.8 CaCl_2_, 1.0 MgCl_2_, 10.0 glucose, and 10.0 HEPES (pH 7.4, adjusted with NaOH). Single CMs were approached under visual control by use of infrared videomicroscopy (S/W-camera CF8/1, Kappa). Recordings were performed in whole-cell current-clamp mode using a patch-clamp amplifier (EPC-9/2, Heka).

### Epifluorescence imaging of intracellular calcium oscillations

Measurement of Ca^2+^-transients was performed after EBs of riPSCs were plated onto fibronectin-coated glass bottom dishes (WPI) on day 20 of differentiation. CMs were loaded with 10 μM Fluo-4 AM including 20% (w/v) Pluronic (both Thermo Scientific) for 30 min at 37°C. After two washing steps using Tyrode solution (mM: 120 NaCl, 5.4 KCl, 1 MgCl_2_, 1.8 CaCl_2_, 5 HEPES, 10 D(+)-glucose, 0.3 L-ascorbic acid), pH 7.4. De-esterification was allowed for 20 min, before imaging (excitation wavelength: 488 nm, emission wavelength: 515 nm) was performed in Tyrode using a Zeiss Axio Observer fluorescence microscope equipped with a Polychrom V (Till), life cell imaging Box (Oko-Lab), EXi Aqua Camera (Q-Imaging) and multi bandpass filter set No. 25 (Zeiss) under the control of Live Acquisition Software (Till). To detect ß-adrenergic and muscarinergic stimulation of Ca^2+^-transients in riPSC-derived CMs, isoprenaline (1 μM) was added to the medium followed by 1 μM carbachol. Analysis of calcium transients was conducted offline using ImageJ.

### Field potential analysis using microelectrode arrays

Extracellular field potentials of riPSC-derived CMs were analyzed using a microelectrode array (MEA) data acquisition system (Multi Channel Systems). EBs on day 16 of differentiation were plated onto fibrin-coated (25 μg/mL) planar-type MEAs of 64 electrodes (TiN, diameter: 30 μm, electrode spacing 200 μm, Multi Channel Systems) and were cultured for two additional days in differentiation medium. Measurements were conducted at baseline and in the presence of isoprenaline (1 μM), quinidine (30 μM) and lidocaine (1 mM). The McRack software (Multi Channel Systems) was used to record and analyze field potentials.

### Statistical analysis

GraphPad Prism software (version 5.03 for Windows; GraphPad Software) was used for statistical analysis. Reported values are means ± SEM. Unless stated otherwise, data were analyzed using the Student’s *t*-test. Statistical significance was assigned as reported individually.

## Results

### Expansion and characterization of rPSCs in feeder-dependent and feeder-free culture

**Fig 1:**
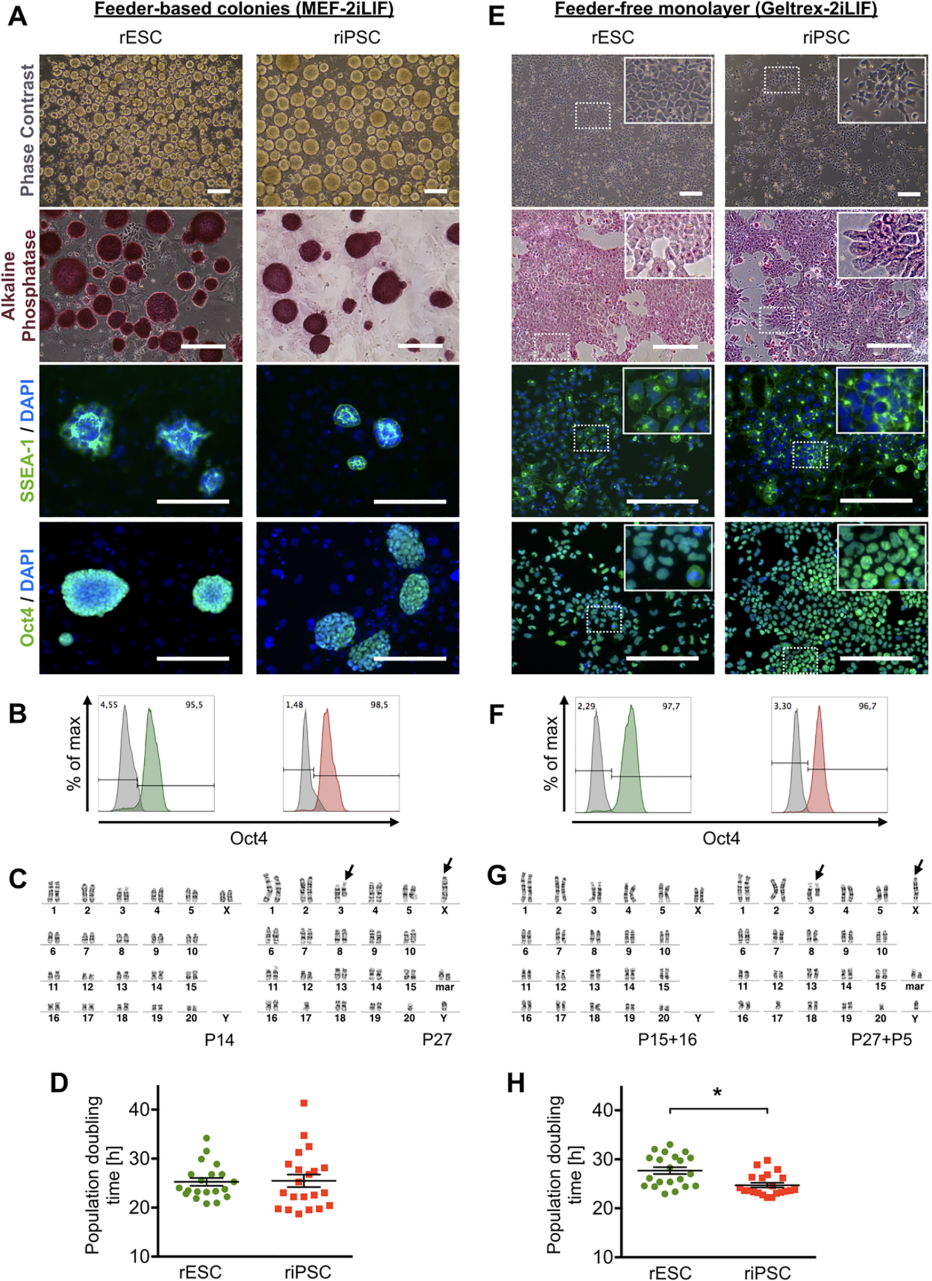
Characterization of undifferentiated rESC and riPSC under feeder-dependent and feeder-free culture conditions. (A) Rat PSCs form floating or loosely attached colonies on mitotically inactivated MEFs (MEF-2iLIF). Both cell types are positive for alkaline phosphatase activity and show SSEA-1 and Oct4 expression. Scale bars: 200 μm. (B) Flow cytometry analyses of both rPSC types for Oct4 expression. (C) Representative diploid karyograms of rPSCs originating from MEF-2iLIF conditions. P indicates the passage number under MEF-2iLIF conditions. rESCs in passage 14 showed a normal female rat karyotype (42, XX). riPSCs in passage 27 presented with an aberrant male karyotype characterized by a translocation between one homolog of chromosome 3 and the X chromosome (arrows) and two marker chromosomes (mar), presumably composed of material from chromosomes 17 and 20 which are missing. (D) No significant difference in population doubling time was detected between rESC and riPSC. Mean ± SEM, n = 20–21, unpaired t-test, P = 0.897. (E) Also in feeder-free monolayer culture (Geltrex-2iLIF), undifferentiated rPSCs express pluripotency markers. Phase contrast images reveal the typical high nucleus-to-cytoplasm ratio of pluripotent stem cells. In addition, cells show alkaline phosphatase activity, and are immuno-positive for SSEA-1 and Oct4. Scale bars: 200 μm. (F) Flow cytometry analysis revealed Oct4^pos^ expression levels comparable to feeder-based cultures for both rPSC types. (G) Representative karyograms of rPSCs after several passages in Geltrex-2iLIF conditions. P indicates the passage number of cells originating from MEF-2iLIF conditions plus additional passages in Geltrex-2iLIF. After 16 passages, rESC still show a normal rat karyotype in the majority of metaphase plates. After 5 passages, riPSCs also showed no differences to the karyotype found under MEF-2iLIF conditions. However, after 18 passages in Geltrex-2IiLIF, the majority of cells showed a tetraploid karyotype (see S1 Fig). (H) Proliferation rates show a significant difference of rESCs versus riPSCs in Geltrex-2iLIF culture. Mean ± SEM, n = 21, unpaired t-test, *P < 0.001.

From colleagues we obtained rESC and riPSC lines from Dark Agouti and Fischer344 rat inbred strains, respectively [20,26]. To confirm rat strain identity, microsatellite genotyping was performed (S1 Fig). Initially, undifferentiated cells were maintained on gamma-irradiated murine embryonic fibroblasts in serum-free N2B27-2iLIF medium (MEF-2iLIF) as previously described [13]. Expansion under these conditions resulted in floating or loosely attached colonies, which were positive for pluripotency markers alkaline phosphatase (AP), stage specific embryonic antigen 1 (SSEA1) and octamer-binding factor 4 (Oct4) (Fig 1A and 1B). Both cell types had a diploid karyotype in 83% of rESC (Passage 14) and 73% of riPSC (Passage 27) metaphases, respectively (Fig 1C). Analyses of later passages revealed a diploid karyotype in 86% of rESCs (Passage 23) and of 84% in riPSCs (Passage 42), indicating chromosomal stability over several passages on feeder cells (S1 Fig). Analysis of population doubling times did not show significant differences between rESC (25.3±0.8 h, n = 20) and riPSC (25.5±1.3 h, n = 21, Fig 1D). With the aim of simplifying culture conditions for convenient up-scaling, in a second step, we adapted undifferentiated rPSCs to feeder-free monolayer cultures on Geltrex-coated culture dishes (Geltrex-2iLIF), as described previously [20]. Both rPSC types grew with a high nucleus-to-cytoplasm ratio, characteristic for rPSC monolayer-cultures, and showed the same expression of pluripotency markers as was observed in MEF-2iLIF conditions (Fig 1E). Effects of long-term monolayer culture on the pluripotent state of cell populations as well as their chromosomal integrity were determined. As evaluated by flow cytometry, expression of Oct4 was robust even in high monolayer passages in both, rESCs and riPSCs (15 and 18 passages, respectively, Fig 1F). Interestingly, rESCs showed a relatively normal karyotype for up to 16 passages with 73% of diploid metaphases, whereas riPSCs had 62% diploid metaphases after 5 monolayer passages (Fig 1G), but were almost completely tetraploid after 18 monolayer passages (S1 Fig). Comparison of proliferative activity under feeder-free monolayer conditions revealed a significantly shorter population doubling time of riPSCs compared to rESCs (24.7±0.5 h vs. 27.7±0.7 h, Fig 1H). A direct correlation between increased passage number and decreased population doubling time was not detected. Therefore, the cause of the observed difference in proliferative activity between the two rPSC types expanded in monolayer conditions remained unclear.

### Ascorbic acid-2-phosphate supplementation robustly enhances cardiac differentiation of rESCs and riPSCs

**Fig 2:**
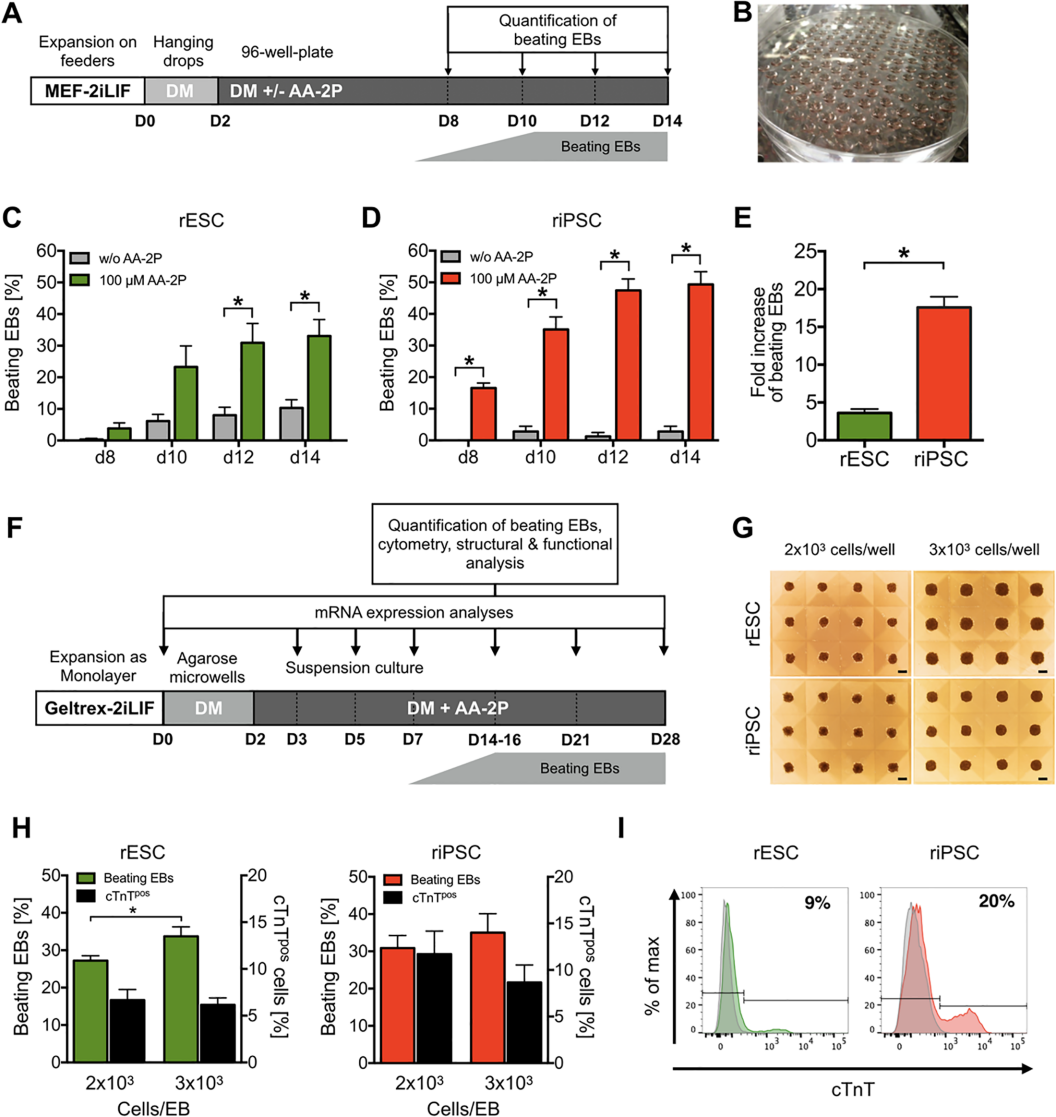
Cardiac differentiation of rPSCs is enhanced by ascorbic acid-2-phosphate and is scalable using agarose microwells. (A) The differentiation protocol started (D0, day 0) with preparation of hanging drops using 3x10^3^ undifferentiated rPSCs (expanded in MEF-2iLIF conditions) per droplet in serum-based differentiation medium (DM). After 2 days (D2), resulting EBs were transferred individually to agarose coated 96-well plates with or without addition of 100 μM AA-2P. Numbers of beating EBs were quantified on day 8, 10, 12 and 14. (B) Cell culture plate with approximately 160 hanging drops. (C,D) Efficiency of cardiac differentiation of rPSC-EBs determined by the emergence of beating EBs over time with and without AA-2P supplementation. Mean ± SEM, (n = 4–10 independent experiments with 48 EBs per biological repetition). (E) Fold increase of beating EB number after of AA-2P supplementation analyzed on day 14. Mean ± SEM, (n = 5–10 independent experiments). Unpaired t-test, *P < 0.05. (F) Protocol for rPSC differentiation expanded in Geltrex-2iLIF conditions using forced aggregation in agarose microwells for two days. Subsequent differentiation was conducted in dynamic suspension culture. (G) rPSC aggregates with different starting cell numbers after 48 h on agarose microwells. Scale bar: 200 μm. (H) Efficiency of cardiac differentiation determined by the quantification of beating EBs on day 14 (left Y-axis). Mean ± SEM, n = 9–26 independent experiments, *P < 0.02. Cardiac differentiation efficiency determined by flow cytometry analysis of cardiac troponin T (cTnT)-stained EB-derived cells on day 14 (right Y-axis). n = 3–7. (I) Exemplary histograms of cTnT-flow cytometry analyses on differentiation day 14. Isotype controls are shown in gray.

First, we tested a directed differentiation protocol, based on EB formation in agarose microwells and temporal modulation of canonical Wnt signaling, that we developed for human PSCs [30]. This led to the formation of stable EBs from rPSCs (S2 Fig), but did not result in contracting CMs. Therefore, we chose classical spontaneous differentiation with hanging drop culture and FCS-based differentiation medium (Fig 2A and 2B). EB formation was induced from feeder-dependent expansion culture using 3x10^3^ undifferentiated rPSCs per drop. We observed that different batches of FCS in the differentiation medium critically influenced cardiac differentiation efficiency (S2 Table and S2 Fig). Therefore, for all described experiments, a pretested batch of FCS (i.e. FCS-3) was used.

Although the supportive effect of ascorbic acid on cardiac differentiation in murine and human PSCs has been reported [31–34], the effects of this agent on rPSC differentiation has not been assessed. Therefore, on day two of differentiation, EBs were cultivated in differentiation medium with or without 100 μM ascorbic acid-2-phosphate (AA-2P). Incubation with AA-2P resulted in improved cardiac differentiation efficiencies in both PSC types (Fig 2C and 2D), however, to a different degree. Notably, without AA-2P, rESCs showed higher cardiac differentiation efficiencies than riPSCs (i.e. 10.3±2.6 vs. 2.8±1.6% on day 14), but displayed a smaller improvement when exposed to AA-2P (33.0±5.2 vs. 49.3±4.0%). Consequently, cardiac differentiation was increased 3.61±0.51-fold in rESC and 17.6±3.18-fold in riPSC by AA-2P (Fig 2E).

To further simplify cardiomyocyte production, we combined the monolayer expansion with a scale-up of the ascorbic acid-supported differentiation process. For this, forced aggregation of rPSCs employing agarose microwells (AMW) was tested to induce EB formation (Fig 2F and S3 Fig). As we have previously shown for mouse and human PSCs, the AMW approach simplifies the differentiation procedure by omitting the labor- and material-intense hanging drop method [30]. Two different cell densities were assessed to generate rPSC-derived EBs, i.e. 2 x 10^3^ and 3 x 10^3^ rPSCs (Fig 2G). After 48 h, AMW-derived aggregates were transferred to dynamic suspension culture (orbital shaker, 60 rpm). In terms of beating EBs on day 14, the higher number of rPSCs per EB was superior for both cell types, with a significant difference for rESCs (Fig 2H). However, EBs produced with less starting cells showed a trend toward a higher proportion of cTnT^pos^ CMs, resulting in higher CM purity for riPSCs (rESCs: 6.7±1.1% vs. 6.2±0.7%; riPSC: 11.7±2.5% vs. 8.7±1.9%). Using 2 x 10^3^ cells per EB, a maximum purity of 9% (rESCs) and 20% (riPSCs) cTnT^pos^ CMs was achieved on day 14 of differentiation (Fig 2I). In contrast to riPSCs in the group with 3 x 10^3^ cells per EB, we observed that individual rESC-EBs were significantly larger already on day 2 (projection area: 49.1±0.9 μm^2^x10^3^ vs. 33.1±1.0 μm^2^x10^3^, S3 Fig). On day 14 of differentiation, this discrepancy was even more pronounced in rESC EBs being 5 times larger than that of riPSC EBs, indicating a higher proliferative activity and a propensity to generate cystic structures (S3 Fig and S1 Video).

### Expression analysis of pluripotency and cardiac markers in differentiating rPSC-EBs

**Fig 3:**
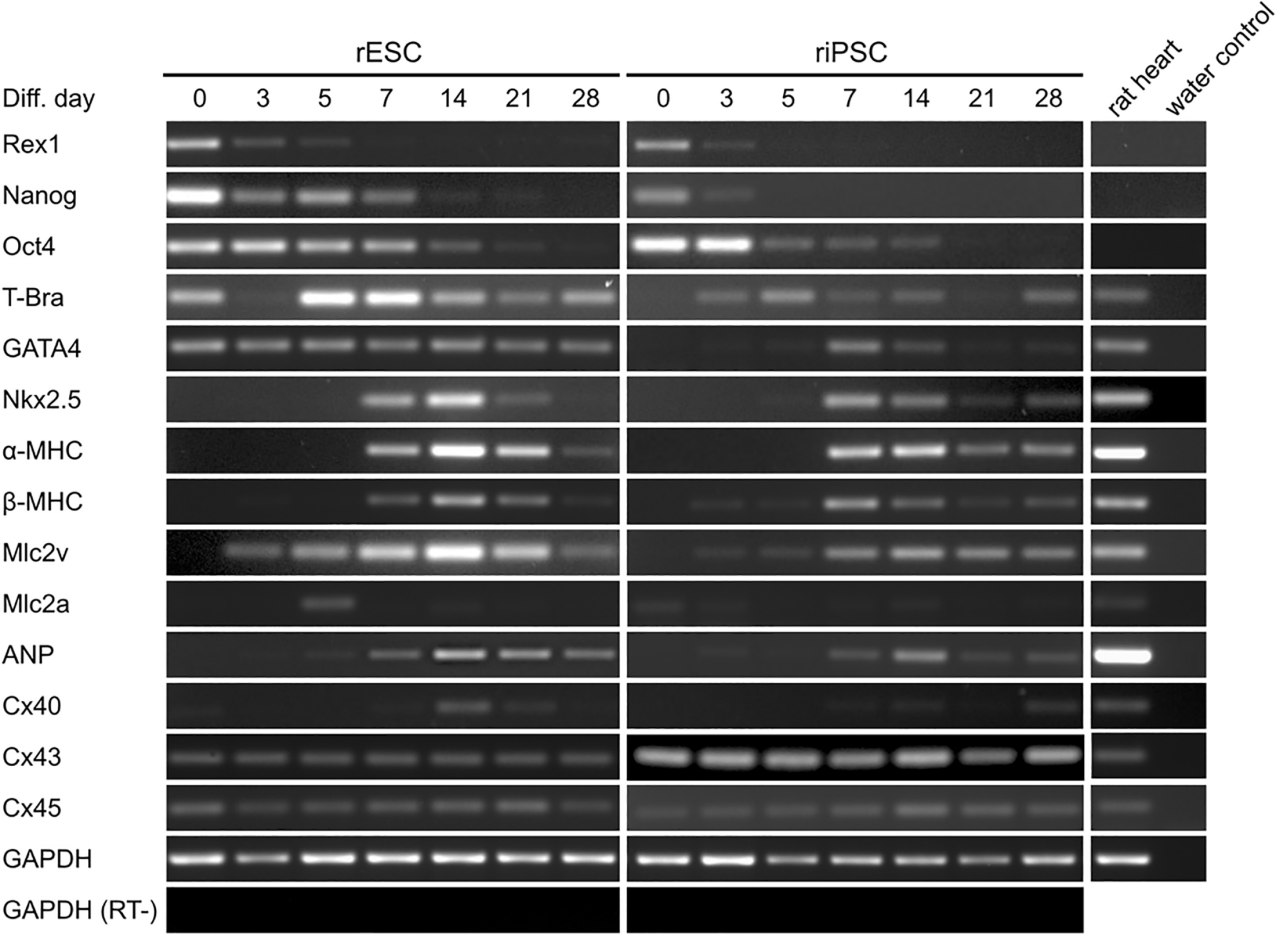
Expression pattern of rPSCs during differentiation. Semiquantitative RT-PCR analyses showing the expression of pluripotency (Rex-1, Nanog, Oct4), mesoderm (T-Bra), CM-specific (GATA4, Nkx2.5, α-MHC, β-MHC, Mlc2v, Mlc2a, ANP) and gap junction (Cx40, Cx43, Cx45) genes in differentiating rESCs and riPSCs derived from AMW-based EB-formation and AA-2P enhanced differentiation in dynamic suspension culture. Expression of Glyceraldehyde-3-phosphate dehydrogenase (GAPDH) served as internal control; water and reverse transcriptase minus (RT-) indicate negative controls.

The differentiation process of rPSCs into functional CMs was analyzed by semi-quantitative reverse transcriptase PCR on undifferentiated rPSC and on days, 3, 5, 7, 14, 21 and 28 of differentiation (Fig 3). Both cell types showed down-regulation of pluripotency-associated transcription factors Rex-1, Nanog and Oct4. In riPSCs, these factors disappeared faster with almost undetectable levels of Rex-1 and Nanog on day 5 and Oct4 on day 28 of differentiation. The expression of the mesendodermal marker T-Bra (Brachyury) was positive in undifferentiated rESCs, consistent with the observation of Chen *et al*. [26], but undetectable in riPSCs. T-Bra was up-regulated during the differentiation process and showed the strongest expression on day 5 in both cell types, whereas less T-Bra expression was detectable at later time points. In addition, we observed an unexpected expression of the cardiac mesodermal marker GATA4 at all time points of differentiating rESCs. In contrast, and as expected, expression of GATA4 was not found in undifferentiated riPSCs and showed a peak expression at day 7. The cardiac mesoderm markers Nkx2.5 as well as α- and β-myosin heavy chain (αMHC and βMHC, resp.) were expressed from day 7 on and reached highest levels of expression on day 7 and 14, respectively. Starting from day 3 of differentiation, an increasing expression of the ventricular marker Mlc2v was observed. Expression of Mlc2a (atrium) was clearly detectable in rESCs on day 5, only. Interestingly, mRNA of gap junction proteins Connexin 43 and 45 (Cx43 and Cx45, resp.) was expressed throughout the differentiation process. We confirmed this for Cx43 also on the protein level by immunofluorescence staining of undifferentiated rPSCs (S4 Fig). As was already described in detail for undifferentiated human iPSCs by Beckmann *et al*., Cx43 was distinctly localized at cell-cell borders of rPSCs [35].

### rPSC-derived cardiomyocytes show distinct cross-striation and the expression of gap junction proteins

**Fig 4:**
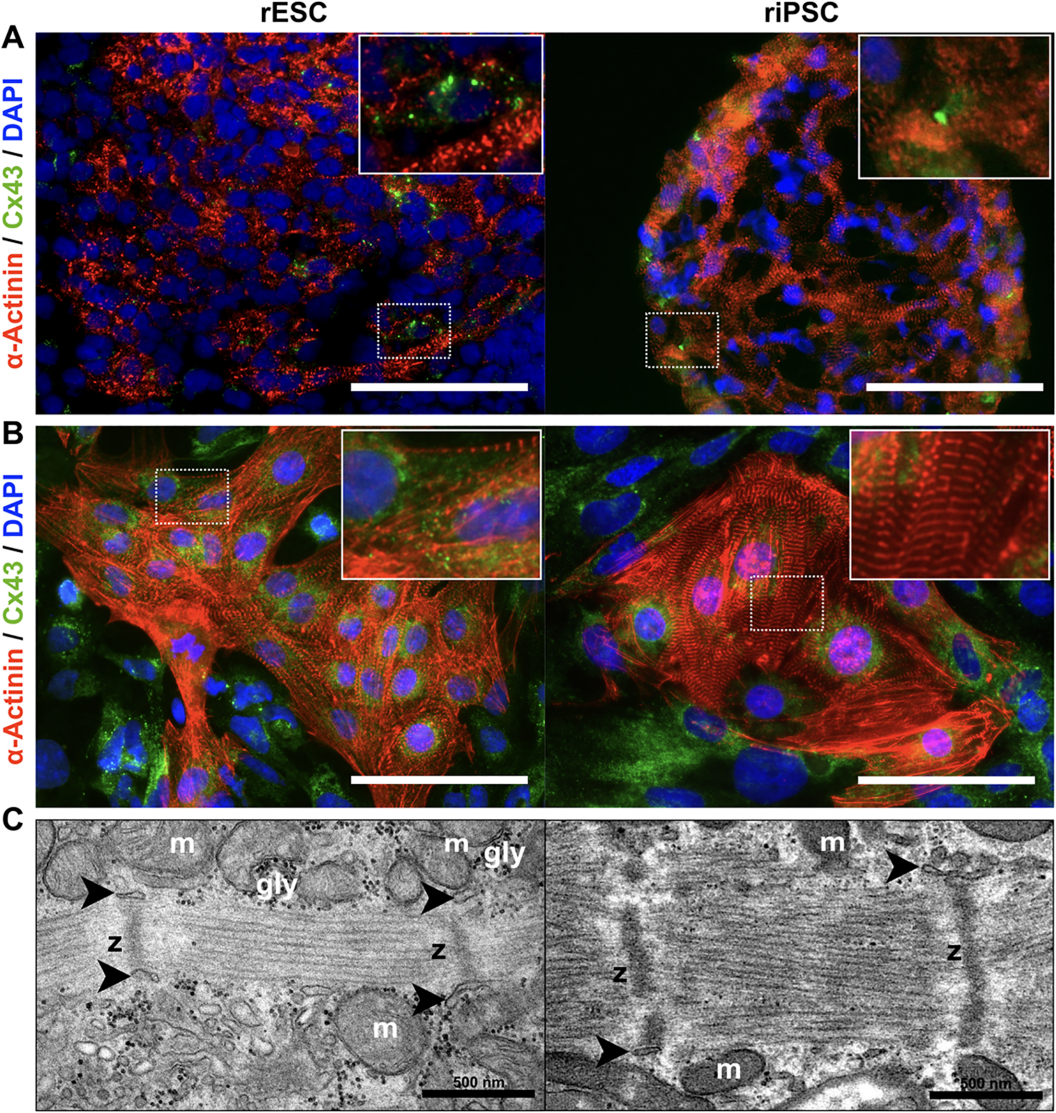
Detection of sarcomeric structures and gap junctions in rPSC-derived cardiomyocytes. (A) Immunofluorescence stainings of cryosections (thickness: 10 μm) of 14 days old beating EBs derived from rESCs and riPSCs differentiated in scalable suspension culture. Sarcomeric α-Actinin and gap junction protein Cx43. Nuclei are counterstained with DAPI. Scale bars: 100 μm. (B) Immunofluorescence staining of sarcomeric α-Actinin and Cx43 on cells after dissociation of EBs (day 14) and re-seeding on fibronectin coated glass-bottom dishes. Scale bars: 100 μm. (C) Transmission electron microscopy images of ultra-thin sections (approx. 70 nm) of EBs on day 14 of differentiation showing distinct Z-bands (z) of myofibrils, abundant mitochondria (m), and dark granules of glycogen (gly) depositions. Arrowheads indicate cisternae of sarcoplasmic reticulum in close proximity to Z-bands. Scale bars: 500 nm.

To identify and localize CM-specific protein expression in rPSC-derived CMs, i.e. sarcomeric and gap junction proteins, immunofluorescence stainings were performed. CMs of both rPSC types showed well-organized sarcomeres in stainings against sarcomeric α-Actinin, cTnT and Titin, both, in cross-sections of floating EBs on day 14 and in adherent cells from dissociated EBs (Fig 4A and 4B and S5 Fig). Cx43 showed marked but weak circumferential expression in α-Actinin-positive cells of EB cryosections. Three days after dissociation and reseeding, Cx43 was detectable within the perinuclear space of CMs, indicating an immature phenotype. Transmission electron microscopy analysis confirmed the CM-specific ultrastructure in cell derivatives of both rPSC types. These included myofibrils with sarcomeres separated by distinct Z-bands, cisternae of sarcoplasmic reticulum in close proximity to the Z-bands, abundant mitochondria, glycogen deposition in the cytoplasm and prominent desmosomes between adjacent cells (Fig 4C and S5 Fig). A prominent circumferential membrane-bound localization of Cx43 and a more pronounced sarcomeric structure was observed at later stages of riPSC-differentiation (day 40), indicating a higher maturation stage of these CMs (S6 Fig).

### Electrophysiology and intracellular Ca^2+^-oscillation of riPSC-derived cardiomyocytes reveal different subtypes and physiological responses to cardio-active drugs

**Fig 5:**
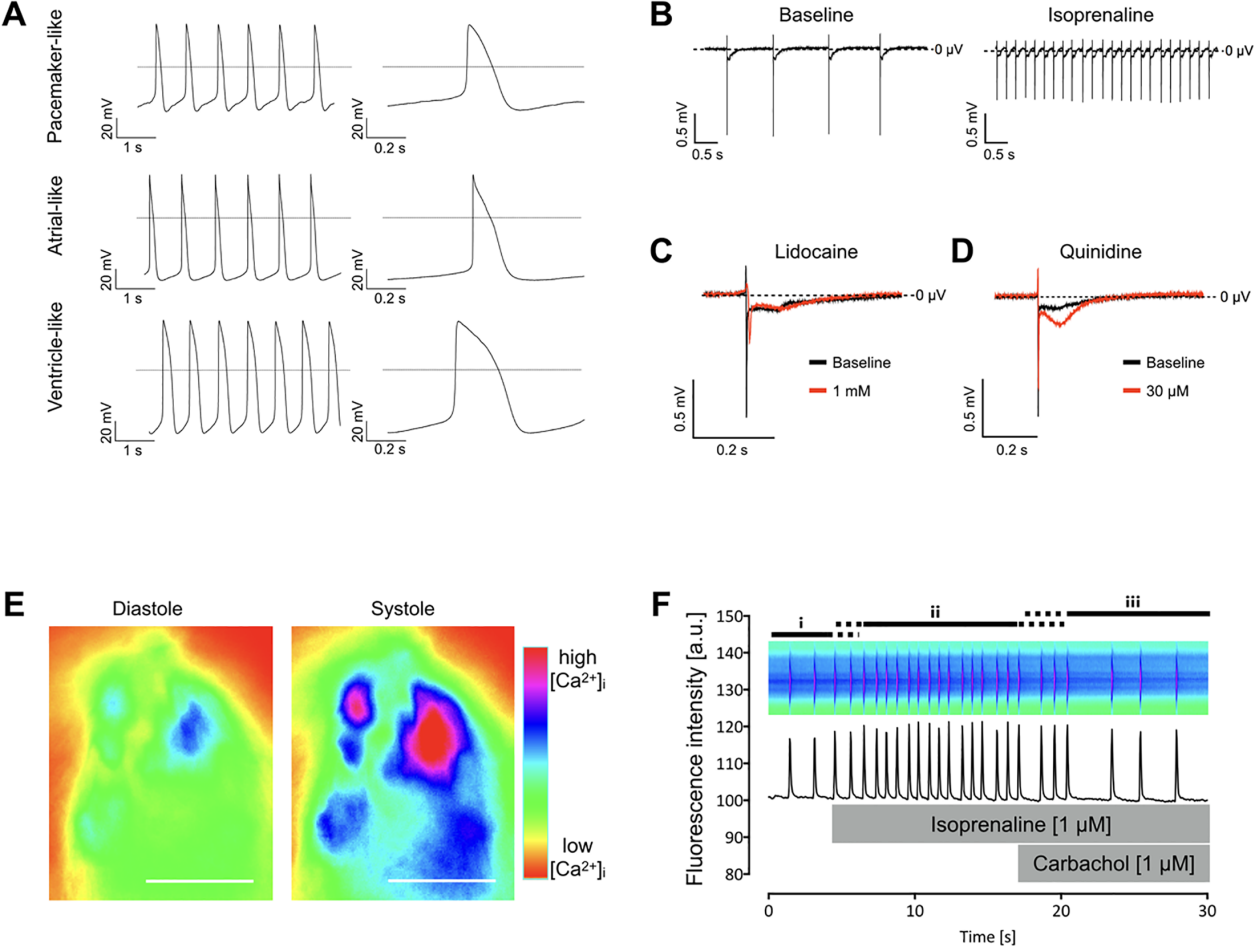
Physiological properties of riPSCs-derived cardiomyocytes. (A) Patch-clamp analysis showing distinct action potential characteristics with pacemaker-, atrial-, and ventricle-like time courses. Horizontal lines show zero potential. (B-D) Extracellular field potential (FP) analyses with microelectrode arrays. (B) Administration of isoprenaline demonstrated physiological drug responsiveness. (C) Application of the sodium channel blocker lidocaine retarded the fast FP component corresponding to the Na^+^-peak. (D) Exposure to the HERG channel inhibitor quinidine increased the amplitude of the plateau phase of FPs. (E) Fluorescence images of riPSC-derived cardiomyocytes loaded with Fluo-4AM. Intracellular [Ca^2+^]_i_ is color-coded, with green to light blue indicating low diastolic [Ca^2+^]_I,_ while violet to red represents high diastolic [Ca2+]_i_, respectively. (F) Representative pseudo-linescan showing spontaneous whole cell [Ca^2+^] transients at baseline (i), isoprenaline-induced β-adrenergic-mediated increase in frequency (ii) and carbachol-induced reduction of beating frequency (iii).

Cardiac action potentials (APs) were recorded using whole cell patch-clamp analysis on spontaneously beating riPSC-CMs on day 20 of differentiation. Based on the time course of APs three different categories of cardiac action potentials were identified, confirming the complete cardiac differentiation capability of riPSCs (Fig 5A). We found pacemaker-like APs with a relatively depolarized membrane potential, slow diastolic depolarization, no plateau phase and relatively low overshoot. Ventricle- and atrial-like APs with a more negative minimum diastolic potential and a higher amplitude were identified by their specific waveforms. Ventricle-like CMs displayed a characteristic rapid depolarization phase and a distinct plateau phase, whereas atrial-like APs showed a fast upstroke with relatively short AP duration. Extracellular field potential (FP) analysis of riPSC-CMs on microelectrode arrays revealed physiological responses to cardio-active drugs (Fig 5B–5D). CMs responded with an increase in beating frequency to β-adrenergic stimulation (Fig 5B), whereas administration of 1 mM lidocaine reduced the FP amplitude and 30 μM quinidine resulted in an increase of the plateau phase of the FP (Fig 5C and 5D). Intracellular Ca^2+^-oscillation in riPSC-CMs was analyzed using epifluorescence microscopy on differentiation day 20 after staining with the calcium-sensitive dye Fluo-4 AM (Fig 5E). Rhythmic Ca^2+^ transients were observed with a marked increase in frequency after β-adrenergic stimulation with 1 μM isoprenaline (Fig 5F). Immediately after co-administration of the muscarinergic receptor agonist carbachol (1 μM), beating frequency decreased, suggesting the physiological coupling of positive and negative inotropic effects mediated by functional β-adrenergic and muscarinergic signaling systems.

## Discussion

Although germline-competent riPSCs are available, there are still no detailed reports on their cardiac differentiation potential. In this study, we assessed a simplified expansion technique and a scalable cardiac differentiation approach in a side-by-side comparison between rESCs and riPSCs. The major findings are: (I) rPSCs can be expanded in Geltrex-2iLIF conditions without losing pluripotency, (II) ascorbic acid 2-phosphate significantly enhances the development of functional CMs in both rPSC types, (III) stable cardiac differentiation is feasible employing agarose microwells to induce EB formation with subsequent dynamic suspension culture, (IV) differentiating rESCs and riPSCs show comparable cardiac gene expression profiles (V) riPSC-CMs display morphological features, physiological properties and pharmacological responses comparable to what is reported for primary rat fetal CMs and rESC-CMs [17]. Therefore, the presented approach may be useful for future myocardial reconstruction studies *in vivo*.

PSC-derived CMs are a promising source for organ-specific *in vitro* disease modeling [36–38], pharmaceutical compound and safety testing [39–41] and reconstructive cell therapies [8,42–45]. Especially the latter has gained tremendous attention, due to promising small and large animal studies that showed substantial restoration after transplantation into ischemic myocardium. Some of these studies were based on xenogeneic models [43,44], whereas other recent reports described allogeneic [46] or even HLA-matched allogeneic [29] situations, respectively. However, immunological, technical and safety related aspects have to be addressed in more detail, before clinical translation of PSC-based myocardial reconstruction can be envisioned. A bottleneck in these transplantation studies is the provision of sufficient numbers of PSC-derived CMs and the costs and feasibility of animal surgery and subsequent monitoring. The demand of transplantable cells increases proportional to the size of the selected animal model, ranging from 3 x 10^4^ in small animal models, such as mice [47] to 1 x 10^9^ PSC-CMs for the left ventricle of small non-human primates [43]. For murine and human ESCs and iPSCs, determinants of cardiac differentiation have been examined for years [48–50] and recent studies report significant improvement in the generation of CMs through modulation of relevant pathways using growth factors and small molecules in staged protocols [51,52]. Furthermore, genetic and metabolic selection procedures resulted in almost pure PSC-CM populations [33,53,54]. Although the rat is of high interest as a pre-clinical model, especially for cardiosurgical reconstructive interventions, until now only one study reports about *in vitro* differentiation of rESCs into functional CMs [17]. However, the application of these CMs to regenerate ischemic myocardium *in vivo* has not been described to date. This might be due to the inability to produce large quantities of transplantable cardiac cells. Hence, we aimed at simplification of culture conditions and optimization of cardiac differentiation efficiency of both available pluripotent stem cell types, i.e. rESCs and riPSCs.

We succeeded in simplifying the expansion culture of rPSCs by adapting undifferentiated cells to feeder-free monolayer culture. This allowed us to significantly reduce workload and to optimize the following steps of the differentiation procedure, which represents a prerequisite for larger scale rPSC-CM production. Both rPSC types showed stable population doubling times and remained Oct4-positive for up to 18 monolayer passages in culture.

Interestingly, the karyotype of the investigated rESC line remained stable under feeder-free conditions, whereas a high frequency of aneuploidies and polyploidies was observed in high passages of feeder-free riPSC cultures. Genomic instability of *in vitro* cultured PSCs has been an issue for a long time and is subject of worldwide ongoing research efforts. In 2011, two studies were published that analyzed a large number of human ESC and iPSC lines [55,56]. Here, 13% to 34% showed an abnormal karyotype with no differences in susceptibility between human ESCs and iPSCs. However, the reasons for structural or numerical abnormalities are still a matter of debate. Some studies report a clear correlation between enzymatic passaging or feeder-free culture and abnormal karyotypes in human PSCs [56,57], whereas others find no link between culture conditions and the occurrence of abnormal events in human PSCs [55,58]. Recently, an underlying molecular mechanism of frequent chromosomal alterations in human PSCs has been elucidated, connecting a decreased expression of serum response factor with the induction of replicative stress resulting in chromosomal condensation defects [59]. Whether this or another mechanism was responsible for observed chromosomal aberrations of riPSCs in the presented study remains speculative, and is a limitation that has to be investigated in more detail. However, we assume that the feeder-free culture supported the proliferative selection of a population within the original riPSC line that already had a complex karyotype. To further elucidate the reasons for genetic instabilities, future studies will have to analyze more independent and genetically stable riPSC lines, as is the case for human PSCs. Although it is still not clear if or to what extent detected genomic abnormalities of PSCs are linked to malignancy, thorough assessment is needed prior to clinical application [60]. In this regard, ESCs and iPSCs of the rat could be a valuable system to study effects of specific aberrations in a preclinical model, next to the tissue-specific regenerative capacity of PSC derivatives.

We then addressed the cardiac differentiation potential of rESCs and riPSCs. To date, protocols for mouse PSCs using stage-specific Activin/Nodal and BMP4 signaling achieve CM purities ranging from 40 to 60% [21,22,51], whereas state-of-the art protocols using temporal modulation of canonical Wnt signaling robustly give rise to >80% CMs for human PSCs, as we and others have shown [24,30,52]. However, since the latter protocol was not successful for rPSCs, we focused on the classic way to induce differentiation by employing the robust hanging drop method together with a switch to FCS-based differentiation medium and withdrawal of LIF and small molecules. Undifferentiated rESCs or riPSCs, which were expanded as colonies on feeders in serum-free LIF/2i medium, were used at a density of 3 x 10^3^ cells per EB. This cell number was determined as optimal for rESC differentiation by Cao *et al*. [17]. Notably, and consistent with other reports, we observed difficulties in aggregating rPSCs to EBs [12,19]. In this respect, we tested several lots of FCS and encountered a high serum-dependent variability in EB formation, which affected cardiac differentiation accordingly. This led us to use a preselected FCS lot for further analyses.

We tested a common enhancer of cardiac differentiation in mice and humans, i.e. ascorbic acid [31,33,34,61]. Since to date its effects on the cardiac differentiation of rPSCs has not been evaluated, we chose to assess it comparatively between rESCs and riPSCs. Although the exact mechanism was not further deciphered in the present study, the literature suggests that collagen synthesis or the proliferation of cardiac progenitor cells contribute to the beneficial effects of ascorbic acid in cardiac differentiation [32,62]. Together with optimized EB starting cell numbers the administration of 100 μM ascorbic acid-2 phosphate (AA-2P) significantly increased the number of spontaneously beating EBs derived from both PSC types. Interestingly, the riPSC line used in this study showed a more pronounced effect compared to that observed in the investigated rESC line. This was due to two independent issues: a better cardiac differentiation efficiency of rESCs already without AA-2P and a more pronounced increase of beating riPSC-EBs in response to AA-2P exposure. These results suggest that although rPSCs require different conditions for stable undifferentiated expansion than mouse and human PSCs, ascorbic acid seems to be a universal enhancer of cardiomyocyte differentiation across species. Encouraged by these results, the development of species-tailored modulation of relevant pathways, e.g. Wnt signaling [52,62], and further process optimization might improve cardiac differentiation efficiency of rPSCs and is currently underway.

The previously described agarose microwell method to induce EB formation in murine and human PSCs was successfully applied to optimize the scalable process [30]. After EB formation of 300 EBs within one step, cultivation was continued in dynamic suspension culture (orbital shaker at 60 rpm). We assume that the slightly reduced cardiac differentiation efficiency compared to the hanging drop method was due to the increased mechanical stress of EBs in suspension culture. However, this drawback was compensated by a considerable reduction of cell culture consumables and effort and will be further improved in future studies. Moreover, the EB formation in agarose microwells can be scaled up to achieve a higher number of EBs and, hence, CMs for subsequent studies.

Characterization of EBs derived from both rPSC types revealed organized expression of CM markers, the presence of distinct sarcomeric structures and organized myofibrils, functional proteins and cellular CM-specific compartments, such as cisternae of the sarcoplasmic reticulum. We observed that early stage rPSC-derived CMs show an immature pattern of Cx43 localization, i.e. a perinuclear, combined with a weak circumferential expression. Consistent with findings in hESC-derived CMs, on day 40 of rPSC differentiation, we observed a more mature phenotype with distinct circumferential gap junctions at the intercellular borders of adjacent CMs [63]. Stimulation approaches mimicking environmental properties found in the native heart, like three-dimensional tissue engineering, which we described for human and murine PSC-derived CMs could further support the maturation of rPSC-CMs [33]. Respective experiments are subject of investigation in our labs. To further determine functionality, riPSC-CMs were also subjected to functional analyses, showing physiological responses to ß-adrenergic and muscarinergic stimulation and to HERG channel inhibition as determined by extracellular field potential analysis and intracellular Ca^2+^ imaging, respectively. In addition, we confirmed that our differentiation protocol generates CM-characteristic AP kinetics (pacemaker-like, atrial-like, ventricle-like), demonstrating the full cardiomyogenic potential of riPSCs. This is comparable to observations in rESCs [17], as well as in mouse and human PSC-derived CMs [33,64]. Further improvement of cardiac differentiation towards distinct CM subtypes will elucidate the feasibility of regenerating specialized myocardial tissue like the ventricle [64] or the sinus node [65].

Interestingly, we detected Cx43 and Cx45 mRNA transcripts not only in differentiating EBs, but also in undifferentiated rESCs and riPSCs. For Cx43 protein, we could also confirm the expression and localization, i.e. on the cellular surface of rPSCs by means of immunofluorescence stainings. Expression of both Connexins, which seem to be involved in intercellular communication during differentiation and cellular reprogramming, was already reported in the context of undifferentiated human and mouse PSCs [35,66,67].

In conclusion, we demonstrate the generation of functional CMs from rESCs and riPSCs. The scalability of the established approach may be used to produce sufficient numbers of rPSC-derived CMs for regenerative approaches after myocardial infarction in a syngeneic or allogeneic rat model. The application might be by means of single cell injection or transplantation of preformed tissue engineered myocardial constructs. We strongly assume that the presented rPSC system will support the establishment of a small animal model for hypothesis testing of a variety of cell-based regenerative approaches. Therefore, this study represents a major step towards pluripotent stem cell-based myocardial repair in an established small animal model without any drawbacks of graft rejection based on interspecies incompatibilities.

## Supporting information

S1 Fig Characterization of undifferentiated rPSCs. (A) Genotyping of rPSCs based on microsatellite markers by PCR. Both rPSC-lines showed the expected rat strain-specific amplification patterns. (B) rESCs of late passage (P23) under MEF-2iLIF conditions still retained a normal female rat Karyotype (42, XX). (C) Normal male karyogram of rat adipose tissue-derived mesenchymal stem cells (rADMSC) in passage 4, which served as founder cells for reprogramming of riPSCs. (D) Representative diploid karyogram of riPSCs in a late passage (P42) under MEF-2iLIF conditions. The aberrations, which were already present in passage 27 (see Fig 1C), were also found here. Chromosomes involved in the translocation t(X;3) are indicated by arrows, mar indicates structurally abnormal marker chromosomes. (E) Representative karyogram of riPSCs after 27 passages under MEF-2iLIF and additional 18 passages under feeder-free Geltrex-2iLIF conditions. In this stage, the majority of cells showed a tetraploid karyotype derived from the aberrant condition found at passage 27. (F) Summarizing table of cytogenetic data. Split passage numbers represent the amount of passages on feeders plus additional passages in feeder-free Geltrex-2iLIF conditions.
(PDF)

S2 Fig Pretesting experiments for cardiac differentiation of rPSCs. (A) A directed cardiac differentiation protocol for human PSCs resulted in stable EBs of rPSCs but did not lead to the development of beating cardiomyocytes. Scale bars: 500 μm. (B) Different lots of fetal calf serum (FCS) critically influence cardiac differentiation efficiency of riPSC-EBs. In direct comparison, FCS-3 showed the best cardiac differentiation potential and was used for all further experiments. Mean ± SEM, n = 3 independent experiments with approx. 48 EBs per repetition.
(PDF)

S3 Fig Embryoid body formation of rESC and riPSC-EBs on agarose microwells and morphological analyses over time. (A) Reusable silicone master (left) and resulting agarose microwell in a 12 well cell culture plate (right). (B) Vertical scatter plot of EB size distribution 48 h after seeding 2x10^3^ or 3x10^3^ rPSCs per agarose microwell. Values are given as cross-sectional projection area from n = 60–180 EBs of two to three independent experiments. Results are reported as mean ± SEM, *P < 0.0001. (C) Phase contrast image of representative EBs on day 14 of differentiation showing significant morphological differences with larger rESC-EBs and partially pronounced cystic structures. Scale bars: 500 μm. (D) Size distribution analysis of day 14 EBs; n = 35–115 EBs of two to three independent experiments, mean ± SEM, *P < 0.0001.
(PDF)

S4 Fig Expression of Connexin 43 in undifferentiated Oct4-positive rPSCs. Expression of Connexin 43 protein (Cx43) was detected by immunofluorescence staining in both Oct4^pos^ rPSC types. Scale bars: 100 μm.
(PDF)

S5 Fig Expression of sarcomeric structures and ultrastructural analysis in rPSC-derived cardiomyocytes. (A,B) Immunofluorescence stainings of EBs-cryosections of day 14 and plated cells for cardiac Troponin T and Titin. Nuclei are stained with DAPI. Scale bars: 100 μm. (C) Transmission electron microscopy images of EB sections. Z-bands (z), (m) mitochondria, (gly) glycogen, (N) nucleus, (J) intercellular junction. Scale bars: 500 nm.
(PDF)

S6 Fig On day 40 of differentiation, riPSC-derived cardiomyocytes show distinct expression of gap junction protein Connexin 43 and sarcomeric proteins α-Actinin, cardiac Troponin T and Titin. Scale bars: 100 μm.
(PDF)

S1 Table Primers and conditions for microsatellite genotyping and semiquantitative RT-PCR. (PDF)

S2 Table Test lots of fetal calf serum. (PDF)

S3 Table Antibodies used for immunofluorescence stainings and flow cytometry. (PDF)

S1 Video Spontaneously contracting embryoid bodies of rESCs and riPSCs on day 14 of differentiation. (MOV)

## References

[1] Doggrell SA, Brown L. Rat models of hypertension, cardiac hypertrophy and failure. Cardiovascular Research. 1998 7;39(1):89–105. 9764192 10.1016/s0008-6363(98)00076-5

[2] Patten RD, Hall-Porter MR. Small Animal Models of Heart Failure: Development of Novel Therapies, Past and Present. Circ Heart Fail. 2009 2 10;2(2):138–44. doi: 10.1161/CIRCHEARTFAILURE.108.839761 19808329 10.1161/CIRCHEARTFAILURE.108.839761

[3] Reinecke H, Zhang M, Bartosek T, Murry C. Survival, Integration, and Differentiation of Cardiomyocyte Grafts: A Study in Normal and Injured Rat Hearts. Circulation. 1999 7 13;100(2):193–202. 10402450 10.1161/01.cir.100.2.193

[4] Min J-Y, Yang Y, Converso KL, Liu L, Huang Q, Morgan JP, et al Transplantation of embryonic stem cells improves cardiac function in postinfarcted rats. J Appl Physiol. 2002 1;92(1):288–96. doi: 10.1152/jappl.2002.92.1.288 11744672 10.1152/jappl.2002.92.1.288

[5] Zimmermann W-H, Didié M, Wasmeier GH, Nixdorff U, Hess A, Melnychenko I, et al Cardiac grafting of engineered heart tissue in syngenic rats. Circulation. 2002 9 24;106(12 Suppl 1):I151–7. 12354725

[6] Laflamme MA, Chen KY, Naumova AV, Muskheli V, Fugate JA, Dupras SK, et al Cardiomyocytes derived from human embryonic stem cells in pro-survival factors enhance function of infarcted rat hearts. Nat Biotechnol. 2007 8 26;25(9):1015–24. doi: 10.1038/nbt1327 17721512 10.1038/nbt1327

[7] Gerbin KA, Yang X, Murry C, Coulombe KLK. Enhanced Electrical Integration of Engineered Human Myocardium via Intramyocardial versus Epicardial Delivery in Infarcted Rat Hearts. Backx PH, editor. PLoS ONE. 2015;10(7):e0131446 doi: 10.1371/journal.pone.0131446 26161513 10.1371/journal.pone.0131446 PMC4498815

[8] Matsuo T, Masumoto H, Tajima S, Ikuno T, Katayama S, Minakata K, et al Efficient long-term survival of cell grafts after myocardial infarction with thick viable cardiac tissue entirely from pluripotent stem cells. Sci Rep. Nature Publishing Group; 2015 11 7;:1–14. 10.1038/srep16842 PMC4653625 26585309

[9] Masumoto H, Nakane T, Tinney JP, Yuan F, Ye F, Kowalski WJ, et al The myocardial regenerative potential of three-dimensional engineered cardiac tissues composed of multiple human iPS cell-derived cardiovascular cell lineages. Sci Rep. Nature Publishing Group; 2016 7 12;6:1–10. 10.1038/srep29933 PMC4951692 27435115

[10] Kadota S, Pabon L, Reinecke H, Murry C. In Vivo Maturation of Human Induced Pluripotent Stem Cell-Derived Cardiomyocytes in Neonatal and Adult Rat Hearts. Stem Cell Reports. 2017 2 14;8(2):278–89. doi: 10.1016/j.stemcr.2016.10.009 28065644 10.1016/j.stemcr.2016.10.009 PMC5311430

[11] Ogasawara T, Okano S, Ichimura H, Kadota S, Tanaka Y, Minami I, et al Impact of extracellular matrix on engraftment and maturation of pluripotent stem cell-derived cardiomyocytes in a rat myocardial infarct model. Sci Rep. Springer US; 2017 8 9;:1–8. 10.1038/s41598-017-09217-x PMC5561148 28819182

[12] Li P, Tong C, Mehrian-Shai R, Jia L, Wu N, Yan Y, et al Germline competent embryonic stem cells derived from rat blastocysts. Cell. 2008 12 26;135(7):1299–310. doi: 10.1016/j.cell.2008.12.006 19109898 10.1016/j.cell.2008.12.006 PMC2735113

[13] Buehr M, Meek S, Blair K, Yang J, Ure J, Silva J, et al Capture of Authentic Embryonic Stem Cells from Rat Blastocysts. Cell. Elsevier Ltd; 2008 12 26;135(7):1287–98. 10.1016/j.cell.2008.12.007 19109897

[14] Liao J, Cui C, Chen S, Ren J, Chen J, Gao Y, et al Generation of induced pluripotent stem cell lines from adult rat cells. Cell Stem Cell. 2009 1 9;4(1):11–5. doi: 10.1016/j.stem.2008.11.013 19097959 10.1016/j.stem.2008.11.013

[15] Li W, Wei W, Zhu S, Zhu J, Shi Y, Lin T, et al Generation of Rat and Human Induced Pluripotent Stem Cells by Combining Genetic Reprogramming and Chemical Inhibitors. Stem Cell. Elsevier Inc; 2009 1 9;4(1):16–9. 10.1016/j.stem.2008.11.014 19097958

[16] Shao Y, Guan Y, Wang L, Qiu Z, Liu M, Chen Y, et al CRISPR/Cas-mediated genome editing in the rat via direct injection of one-cell embryos. Nat Protoc. Nature Publishing Group; 2014 9 25;9(10):2493–512. 10.1038/nprot.2014.171 25255092

[17] Cao N, Liao J, Liu Z, Zhu W, Wang J, Liu L, et al In vitro differentiation of rat embryonic stem cells into functional cardiomyocytes. Cell Res. Nature Publishing Group; 2011 3 22;21(9):1316–31. 10.1038/cr.2011.48 PMC3193466 21423272

[18] Blair K, Leitch HG, Mansfield W, Dumeau C-É, Humphreys P, Smith AG. Culture parameters for stable expansion, genetic modification and germline transmission of rat pluripotent stem cells. Biology Open. Company of Biologists; 2012 1 15;1(1):58–65. 10.1242/bio.2011029 PMC3507162 23213369

[19] Liskovykh M, Chuykin I, Ranjan A, Safina D, Popova E, Tolkunova E, et al Derivation, Characterization, and Stable Transfection of Induced Pluripotent Stem Cells from Fischer344 Rats. Milstone DS, editor. PLoS ONE. 2011 11 4;6(11):e27345 doi: 10.1371/journal.pone.0027345 22076153 10.1371/journal.pone.0027345 PMC3208629

[20] Merkl C, Saalfrank A, Riesen N, Kühn R, Pertek A, Eser S, et al Efficient Generation of Rat Induced Pluripotent Stem Cells Using a Non-Viral Inducible Vector. Pant AB, editor. PLoS ONE. Public Library of Science; 2013 1 31;8(1):e55170. 10.1371/journal.pone.0055170 PMC3561372 23383095

[21] Kattman SJ, Witty AD, Gagliardi M, Dubois NC, Niapour M, Hotta A, et al Stage-specific optimization of activin/nodal and BMP signaling promotes cardiac differentiation of mouse and human pluripotent stem cell lines. Cell Stem Cell. 2011 2 4;8(2):228–40. doi: 10.1016/j.stem.2010.12.008 21295278 10.1016/j.stem.2010.12.008

[22] Hartman ME, Librande JR, Medvedev IO, Ahmad RN, Moussavi-Harami F, Gupta PP, et al An Optimized and Simplified System of Mouse Embryonic Stem Cell Cardiac Differentiation for the Assessment of Differentiation Modifiers. Barbuti A, editor. PLoS ONE. 2014 3 25;9(3):e93033–12. doi: 10.1371/journal.pone.0093033 24667642 10.1371/journal.pone.0093033 PMC3965510

[23] Lian X, Hsiao C, Wilson G, Zhu K, Hazeltine LB, Azarin SM, et al Robust cardiomyocyte differentiation from human pluripotent stem cells via temporal modulation of canonical Wnt signaling. Proceedings of the National Academy of Sciences. National Acad Sciences; 2012 7 3;109(27):E1848–57. 10.1073/pnas.1200250109 PMC3390875 22645348

[24] Kempf H, Olmer R, Kropp C, Rückert M, Jara-Avaca M, Robles-Diaz D, et al Controlling expansion and cardiomyogenic differentiation of human pluripotent stem cells in scalable suspension culture. Stem Cell Reports. 2014 12 9;3(6):1132–46. doi: 10.1016/j.stemcr.2014.09.017 25454631 10.1016/j.stemcr.2014.09.017 PMC4264033

[25] Wendel JS, Ye L, Tao R, Zhang J, Zhang J, Kamp TJ, et al Functional Effects of a Tissue-Engineered Cardiac Patch From Human Induced Pluripotent Stem Cell-Derived Cardiomyocytes in a Rat Infarct Model. Stem Cells Translational Medicine. 2015 9 14;4(11):1324–32. doi: 10.5966/sctm.2015-0044 26371342 10.5966/sctm.2015-0044 PMC4622407

[26] Chen Y, Blair K, Smith A. Robust Self-Renewal of Rat Embryonic Stem Cells Requires Fine-Tuning of Glycogen Synthase Kinase-3 Inhibition. Stem Cell Reports. The Authors; 2013 9 10;1(3):209–17. 10.1016/j.stemcr.2013.07.003 PMC3849254 24319657

[27] Seabright M. A rapid banding technique for human chromosomes. The Lancet. 1971 10 30;2(7731):971–2. 10.1016/s0140-6736(71)90287-x 4107917

[28] Hamta A, Adamovic T, Samuelson E, Helou K, Behboudi A, Levan G. Chromosome ideograms of the laboratory rat (Rattus norvegicus) based on high-resolution banding, and anchoring of the cytogenetic map to the DNA sequence by FISH in sample chromosomes. Cytogenet Genome Res. 2006;115(2):158–68. doi: 10.1159/000095237 17065798 10.1159/000095237

[29] Kawamura T, Miyagawa S, Fukushima S, Maeda A, Kashiyama N, Kawamura A, et al Cardiomyocytes Derived from MHC-Homozygous Induced Pluripotent Stem Cells Exhibit Reduced Allogeneic Immunogenicity in MHC-Matched Non-human Primates. Stem Cell Reports. 2016 3 8;6(3):312–20. doi: 10.1016/j.stemcr.2016.01.012 26905198 10.1016/j.stemcr.2016.01.012 PMC4788782

[30] Dahlmann J, Kensah G, Kempf H, Skvorc D, Gawol A, Elliott DA, et al The use of agarose microwells for scalable embryoid body formation and cardiac differentiation of human and murine pluripotent stem cells. Biomaterials. Elsevier Ltd; 2013 1 12;:1–9. 10.1016/j.biomaterials.2012.12.024 23332176

[31] Takahashi T. Ascorbic Acid Enhances Differentiation of Embryonic Stem Cells Into Cardiac Myocytes. Circulation. 2003 3 31;107(14):1912–6. doi: 10.1161/01.CIR.0000064899.53876.A3 12668514 10.1161/01.CIR.0000064899.53876.A3

[32] Cao N, Liu Z, Chen Z, Wang J, Chen T, Zhao X, et al Ascorbic acid enhances the cardiac differentiation of induced pluripotent stem cells through promoting the proliferation of cardiac progenitor cells. Cell Res. 2011 12 6;22(1):219–36. doi: 10.1038/cr.2011.195 22143566 10.1038/cr.2011.195 PMC3351910

[33] Kensah G, Lara AR, Dahlmann J, Zweigerdt R, Schwanke K, Hegermann J, et al Murine and human pluripotent stem cell-derived cardiac bodies form contractile myocardial tissue in vitro. European Heart Journal. The Oxford University Press; 2012 10 26;34(15):ehs349–1146. 10.1093/eurheartj/ehs349 23103664

[34] Passier R, Oostwaard DW-V, Snapper J, Kloots J, Hassink RJ, Kuijk E, et al Increased Cardiomyocyte Differentiation from Human Embryonic Stem Cells in Serum-Free Cultures. Stem Cells. 2005 6;23(6):772–80. doi: 10.1634/stemcells.2004-0184 15917473 10.1634/stemcells.2004-0184

[35] Beckmann A, Schubert M, Hainz N, Haase A, Martin U, Tschernig T, et al Ultrastructural demonstration of Cx43 gap junctions in induced pluripotent stem cells from human cord blood. Histochemistry and Cell Biology. Springer Berlin Heidelberg; 2016 7 23;:1–9. 10.1007/s00418-016-1469-9 27456332

[36] Moretti A, Bellin M, Welling A, Jung CB, Lam JT, Bott-Flügel L, et al Patient-specific induced pluripotent stem-cell models for long-QT syndrome. N Engl J Med. 2010 10 7;363(15):1397–409. doi: 10.1056/NEJMoa0908679 20660394 10.1056/NEJMoa0908679

[37] Wang G, McCain ML, Yang L, He A, Pasqualini FS, Agarwal A, et al Modeling the mitochondrial cardiomyopathy of Barth syndrome with induced pluripotent stem cell and heart-on-chip technologies. Nat Med. 2014 6;20(6):616–23. doi: 10.1038/nm.3545 24813252 10.1038/nm.3545 PMC4172922

[38] Veerman CC, Mengarelli I, Guan K, Stauske M, Barc J, Tan HL, et al hiPSC-derived cardiomyocytes from Brugada Syndrome patients without identified mutations do not exhibit clear cellular electrophysiological abnormalities. Sci Rep. Nature Publishing Group; 2016 7 26;:1–10. 10.1038/srep30967 PMC4971529 27485484

[39] Bergström G, Christoffersson J, Schwanke K, Zweigerdt R, Mandenius C-F. Stem cell derived in vivo-like human cardiac bodies in a microfluidic device for toxicity testing by beating frequency imaging. Lab Chip. Royal Society of Chemistry; 2015 7 8;15(15):3242–9. 10.1039/c5lc00449g 26135270

[40] Mannhardt I, Breckwoldt K, Letuffe-Breniere D, Schaaf S, Schulz H, Neuber C, et al Human Engineered Heart Tissue: Analysis of Contractile Force. Stem Cell Reports. 2016 7 12;7(1):29–42. doi: 10.1016/j.stemcr.2016.04.011 27211213 10.1016/j.stemcr.2016.04.011 PMC4944531

[41] Burridge PW, Li YF, Matsa E, Wu H, Ong S-G, Sharma A, et al Human induced pluripotent stem cell–derived cardiomyocytes recapitulate the predilection of breast cancer patients to doxorubicin-induced cardiotoxicity. Nat Med. 2016 4 18;22(5):547–56. doi: 10.1038/nm.4087 27089514 10.1038/nm.4087 PMC5086256

[42] Masumoto H, Ikuno T, Takeda M, Fukushima H, Marui A, Katayama S, et al Human iPS cell-engineered cardiac tissue sheets with cardiomyocytes and vascular cells for cardiac regeneration. Sci Rep. 2014 10 22;4:6716 doi: 10.1038/srep06716 25336194 10.1038/srep06716 PMC4205838

[43] Chong JJH, Yang X, Don CW, Minami E, Liu Y-W, Weyers JJ, et al Human embryonic-stem-cell-derived cardiomyocytes regenerate non-human primate hearts. Nature. 2014 4 30. 10.1038/nature13233 PMC4154594 24776797

[44] Shiba Y, Filice D, Fernandes S, Minami E, Dupras SK, Biber BV, et al Electrical Integration of Human Embryonic Stem Cell-Derived Cardiomyocytes in a Guinea Pig Chronic Infarct Model. J Cardiovasc Pharmacol Ther. 2014 6 17;19(4):368–81. doi: 10.1177/1074248413520344 24516260 10.1177/1074248413520344 PMC4127378

[45] Funakoshi S, Miki K, Takaki T, Okubo C, Hatani T, Chonabayashi K, et al Enhanced engraftment, proliferation, and therapeutic potential in heart using optimized human iPSC-derived cardiomyocytes. Sci Rep. Nature Publishing Group; 2015 12 16;6:1–14. 10.1038/srep19111 PMC4705488 26743035

[46] Shiba Y, Gomibuchi T, Seto T, Wada Y, Ichimura H, Tanaka Y, et al Allogeneic transplantation of iPS cell-derived cardiomyocytes regenerates primate hearts. Nature. 2016 10 20;538(7625):388–91. doi: 10.1038/nature19815 27723741 10.1038/nature19815

[47] Kolossov E, Bostani T, Roell W, Breitbach M, Pillekamp F, Nygren JM, et al Engraftment of engineered ES cell-derived cardiomyocytes but not BM cells restores contractile function to the infarcted myocardium. Journal of Experimental Medicine. 2006 9 21;203(10):2315–27. doi: 10.1084/jem.20061469 16954371 10.1084/jem.20061469 PMC2118112

[48] Wobus AM, Kaomei G, Shan J, Wellner MC, Rohwedel J, Ji Guanju, et al Retinoic acid accelerates embryonic stem cell-derived cardiac differentiation and enhances development of ventricular cardiomyocytes. J Mol Cell Cardiol. 1997 6;29(6):1525–39. 9220339 10.1006/jmcc.1997.0433

[49] Mauritz C, Schwanke K, Reppel M, Neef S, Katsirntaki K, Maier LS, et al Generation of Functional Murine Cardiac Myocytes From Induced Pluripotent Stem Cells. Circulation. 2008 7 28;118(5):507–17. doi: 10.1161/CIRCULATIONAHA.108.778795 18625890 10.1161/CIRCULATIONAHA.108.778795

[50] Kehat I. Human embryonic stem cells can differentiate into myocytes with structural and functional properties of cardiomyocytes. Journal of Clinical Investigation. 2001 8 1;108(3):407–14. doi: 10.1172/JCI12131 11489934 10.1172/JCI12131 PMC209357

[51] Kokkinopoulos I, Ishida H, Saba R, Coppen S, Suzuki K, Yashiro K. Cardiomyocyte differentiation from mouse embryonic stem cells using a simple and defined protocol. Dev Dyn. 2016 2 1;245(2):157–65. doi: 10.1002/dvdy.24366 26515123 10.1002/dvdy.24366

[52] Lian X, Zhang J, Azarin SM, Zhu K, Hazeltine LB, Bao X, et al Directed cardiomyocyte differentiation from human pluripotent stem cells by modulating Wnt/β-catenin signaling under fully defined conditions. Nat Protoc. 2012 12 20;8(1):162–75. doi: 10.1038/nprot.2012.150 23257984 10.1038/nprot.2012.150 PMC3612968

[53] Klug MG, Soonpaa MH, Koh GY, Field LJ. Genetically selected cardiomyocytes from differentiating embronic stem cells form stable intracardiac grafts. Journal of Clinical Investigation. 1996 7 1;98(1):216–24. doi: 10.1172/JCI118769 8690796 10.1172/JCI118769 PMC507419

[54] Tohyama S, Hattori F, Sano M, Hishiki T, Nagahata Y, Matsuura T, et al Distinct metabolic flow enables large-scale purification of mouse and human pluripotent stem cell-derived cardiomyocytes. Cell Stem Cell. 2013 1 3;12(1):127–37. doi: 10.1016/j.stem.2012.09.013 23168164 10.1016/j.stem.2012.09.013

[55] Taapken SM, Nisler BS, Newton MA, Sampsell-Barron TL, Leonhard KA, McIntire EM, et al Karyotypic abnormalities in human induced pluripotent stem cells and embryonic stem cells. Nat Biotechnol. Nature Research; 2011 4 1;29(4):313–4. 10.1038/nbt.1835 21478842

[56] Amps K, Andrews PW, Anyfantis G, Armstrong L, Avery S, Baharvand H, et al Screening ethnically diverse human embryonic stem cells identifies a chromosome 20 minimal amplicon conferring growth advantage. Nat Biotechnol. 2011 11 27;29(12):1132–44. doi: 10.1038/nbt.2051 22119741 10.1038/nbt.2051 PMC3454460

[57] Catalina P, Montes R, Ligero G, Sanchez L, la Cueva de T, Bueno C, et al Human ESCs predisposition to karyotypic instability: Is a matter of culture adaptation or differential vulnerability among hESC lines due to inherent properties? Mol Cancer. 2008;7(1):76–9. 18834512 10.1186/1476-4598-7-76 PMC2567976

[58] Thomson A, Wojtacha D, Hewitt Z, Priddle H, Sottile V, Di Domenico A, et al Human Embryonic Stem Cells Passaged Using Enzymatic Methods Retain a Normal Karyotype and Express CD30. Cloning and Stem Cells. 2008 3;10(1):89–106. doi: 10.1089/clo.2007.0072 18241127 10.1089/clo.2007.0072

[59] Lamm N, Ben-David U, Golan-Lev T, Storchová Z, Benvenisty N, Kerem B. Genomic Instability in Human Pluripotent Stem Cells Arises from Replicative Stress and Chromosome Condensation Defects. Cell Stem Cell. 2016 2 4;18(2):253–61. doi: 10.1016/j.stem.2015.11.003 26669899 10.1016/j.stem.2015.11.003

[60] Peterson SE, Loring JF. Genomic instability in pluripotent stem cells: implications for clinical applications. J Biol Chem. American Society for Biochemistry and Molecular Biology; 2014 2 21;289(8):4578–84. 10.1074/jbc.R113.516419 PMC3931019 24362040

[61] Sato H, Takahashi M, Ise H, Yamada A, Hirose S-I, Tagawa Y-I, et al Collagen synthesis is required for ascorbic acid-enhanced differentiation of mouse embryonic stem cells into cardiomyocytes. Biochemical and Biophysical Research Communications. 2006 3 31;342(1):107–12. doi: 10.1016/j.bbrc.2006.01.116 16480687 10.1016/j.bbrc.2006.01.116

[62] Ivanyuk D, Budash G, Zheng Y, Gaspar JA, Chaudhari U, Fatima A, et al Ascorbic Acid-Induced Cardiac Differentiation of Murine Pluripotent Stem Cells: Transcriptional Profiling and Effect of a Small Molecule Synergist of Wnt/β-Catenin Signaling Pathway. Cell Physiol Biochem. 2015;36(2):810–30. doi: 10.1159/000430140 26021268 10.1159/000430140

[63] Lundy SD, Zhu W-Z, Regnier M, Laflamme MA. Structural and Functional Maturation of Cardiomyocytes Derived from Human Pluripotent Stem Cells. Stem Cells and Development. 2013 7 15;22(14):1991–2002. doi: 10.1089/scd.2012.0490 23461462 10.1089/scd.2012.0490 PMC3699903

[64] Chen Z, Xian W, Bellin M, Dorn T, Tian Q, Goedel A, et al Subtype-specific promoter-driven action potential imaging for precise disease modelling and drug testing in hiPSC-derived cardiomyocytes. European Heart Journal. 2016 6 16;:ehw189–10. 10.1093/eurheartj/ehw189 PMC5381588 28182242

[65] Jung JJ, Husse B, Rimmbach C, Krebs S, Stieber J, Steinhoff G, et al Programming and isolation of highly pure physiologically and pharmacologically functional sinus-nodal bodies from pluripotent stem cells. Stem Cell Reports. 2014 5 6;2(5):592–605. doi: 10.1016/j.stemcr.2014.03.006 24936448 10.1016/j.stemcr.2014.03.006 PMC4050488

[66] Oyamada M, Takebe K, Endo A, Hara S, Oyamada Y. Connexin expression and gap-junctional intercellular communication in ES cells and iPS cells. Front Pharmacol. 2013;4:85 doi: 10.3389/fphar.2013.00085 23840189 10.3389/fphar.2013.00085 PMC3699729

[67] Ke Q, Li L, Cai B, Liu C, Yang Y, Gao Y, et al Connexin 43 is involved in the generation of human-induced pluripotent stem cells. Human Molecular Genetics. 2013 2 18;22(11):2221–33. doi: 10.1093/hmg/ddt074 23420013 10.1093/hmg/ddt074

