# Supplementary material for: Generation of functional cardiomyocytes from rat embryonic and induced pluripotent stem cells using feeder-free expansion and differentiation in suspension culture
Source: PLoS One. 2018 Mar 7;13(3):e0192652. doi: 10.1371/journal.pone.0192652 (PMC5841662; doi:10.1371/journal.pone.0192652)
Supplement: S1 Fig — (A) Genotyping of rPSCs based on microsatellite markers by PCR. Both rPSC-lines showed the expected rat strain-specific amplification patterns. (B) rESCs of late passage (P23) under MEF-2iLIF conditions still retained a normal female rat Karyotype (42, XX). (C) Normal male karyogram of rat adipose tissue-derived mesenchymal stem cells (rADMSC) in passage 4, which served as founder cells for reprogramming of riPSCs. (D) Representative diploid karyogram of riPSCs in a late passage (P42) under MEF-2iLIF conditions. The aberrations, which were already present in passage 27 (see Fig 1C), were also found here. Chromosomes involved in the translocation t(X;3) are indicated by arrows, mar indicates structurally abnormal marker chromosomes. (E) Representative karyogram of riPSCs after 27 passages under MEF-2iLIF and additional 18 passages under feeder-free Geltrex-2iLIF conditions. In this stage, the majority of cells showed a tetraploid karyotype derived from the aberrant condition found at passage 27. (F) Summarizing table of cytogenetic data. Split passage numbers represent the amount of passages on feeders plus additional passages in feeder-free Geltrex-2iLIF conditions. (PDF) [file pone.0192652.s001.pdf]

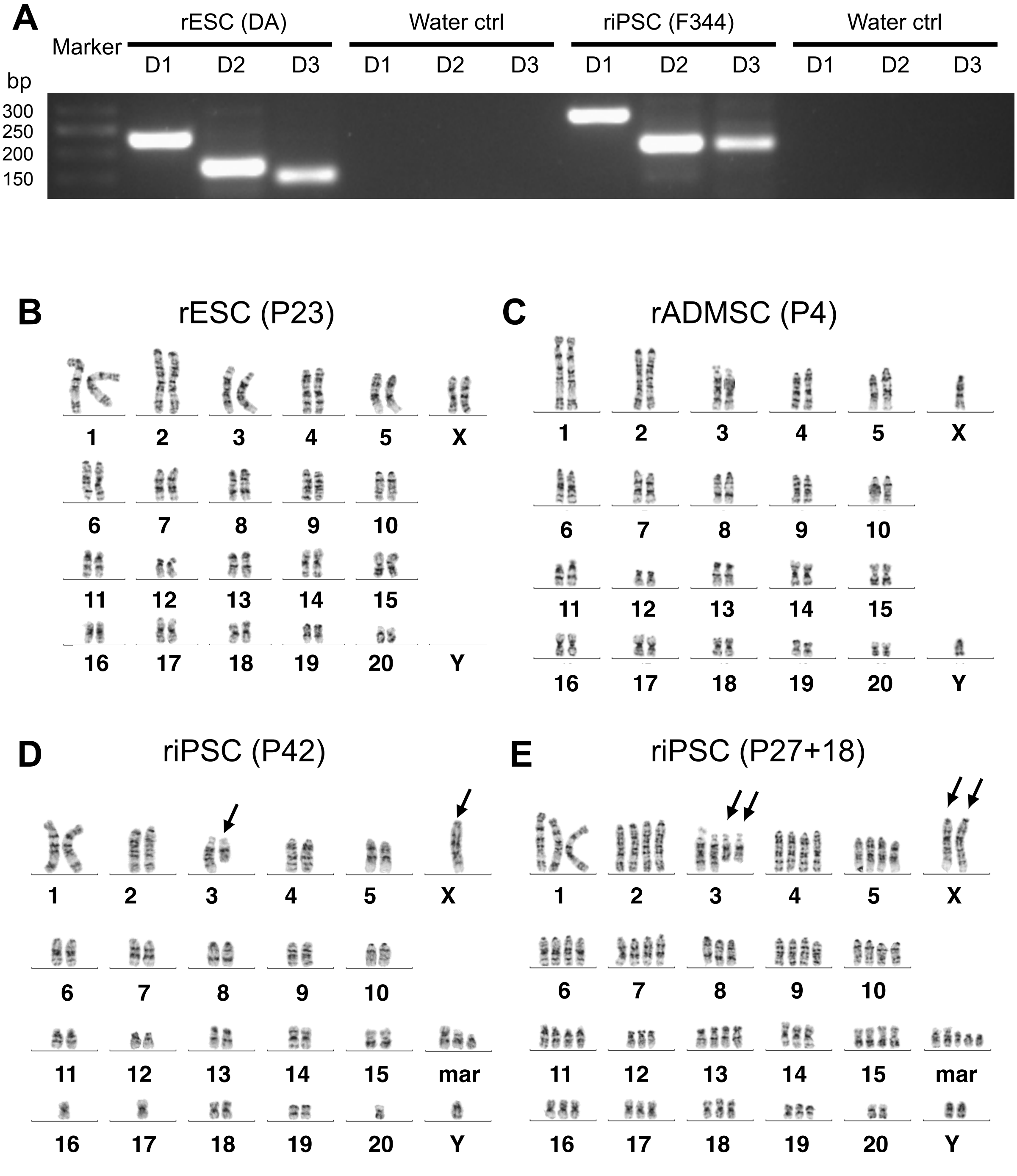

F

Rat strain	Cell type	Gender	Culture system	Passage number	% of diploid metaphases	% of tetraploid metaphases
**Dark Agouti**	rESC	female	MEF-2iLIF	P14	83	17
**Dark Agouti**	rESC	female	MEF-2iLIF	P23	86	14
**Dark Agouti**	rESC	female	Geltrex-2iLIF	P15+16	73	27
**Fischer 344**	rADMSC	male	MEM/10% FCS	P4	96	4
**Fischer 344**	riPSC	male	MEF-2iLIF	P27	73	27
**Fischer 344**	riPSC	male	MEF-2iLIF	P42	84	16
**Fischer 344**	riPSC	male	Geltrex-2iLIF	P27+5	62	38
**Fischer 344**	riPSC	male	Geltrex-2iLIF	P27+18	5	95
