# Supplementary figures and images for: Generation of functional cardiomyocytes from rat embryonic and induced pluripotent stem cells using feeder-free expansion and differentiation in suspension culture
Source: PLoS One. 2018 Mar 7;13(3):e0192652. doi: 10.1371/journal.pone.0192652 (PMC5841662; doi:10.1371/journal.pone.0192652)

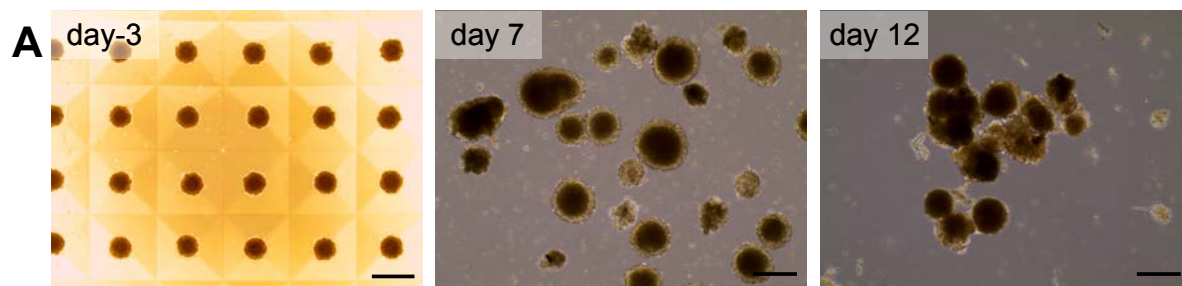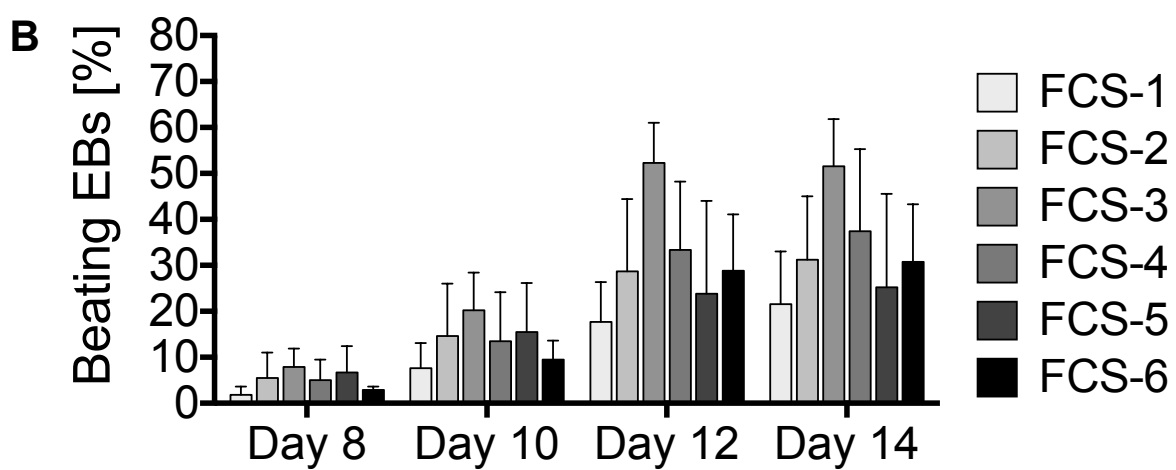

Supplement: S2 Fig — (A) A directed cardiac differentiation protocol for human PSCs resulted in stable EBs of rPSCs but did not lead to the development of beating cardiomyocytes. Scale bars: 500 μm. (B) Different lots of fetal calf serum (FCS) critically influence cardiac differentiation efficiency of riPSC-EBs. In direct comparison, FCS-3 showed the best cardiac differentiation potential and was used for all further experiments. Mean ± SEM, n = 3 independent experiments with approx. 48 EBs per repetition. (PDF) [file pone.0192652.s002.pdf]

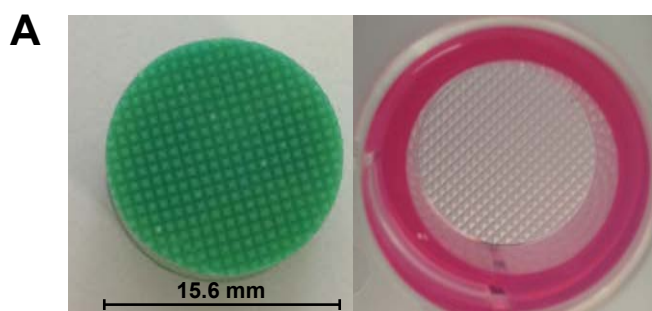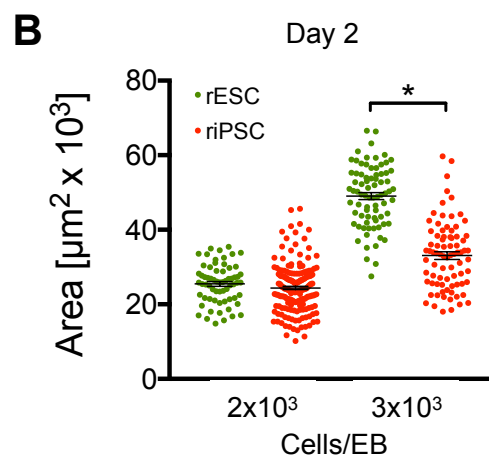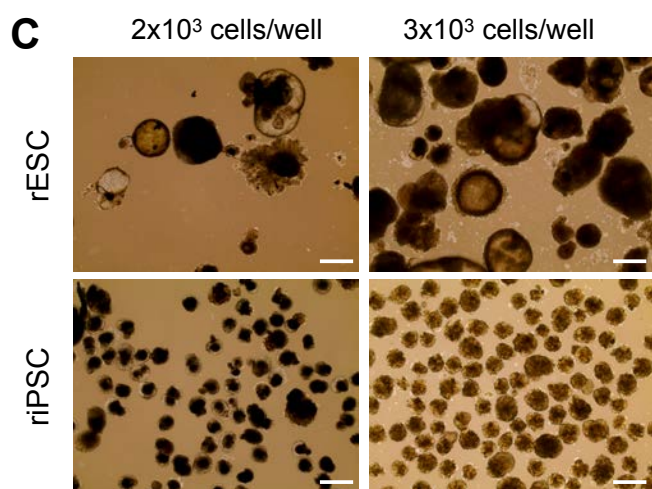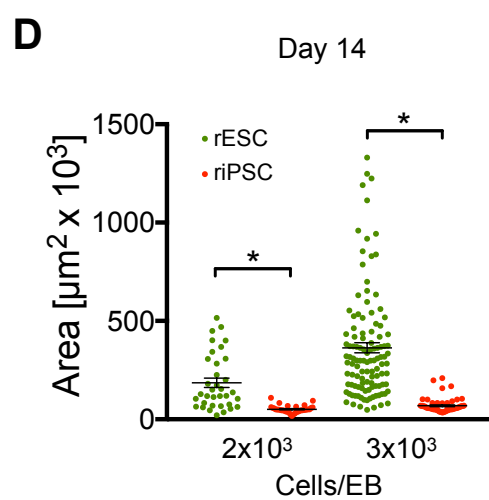

Supplement: S3 Fig — (A) Reusable silicone master (left) and resulting agarose microwell in a 12 well cell culture plate (right). (B) Vertical scatter plot of EB size distribution 48 h after seeding 2x10^3^ or 3x10^3^ rPSCs per agarose microwell. Values are given as cross-sectional projection area from n = 60–180 EBs of two to three independent experiments. Results are reported as mean ± SEM, *P < 0.0001. (C) Phase contrast image of representative EBs on day 14 of differentiation showing significant morphological differences with larger rESC-EBs and partially pronounced cystic structures. Scale bars: 500 μm. (D) Size distribution analysis of day 14 EBs; n = 35–115 EBs of two to three independent experiments, mean ± SEM, *P < 0.0001. (PDF) [file pone.0192652.s003.pdf]

Oct4 / Cx43 / DAPI

rESC

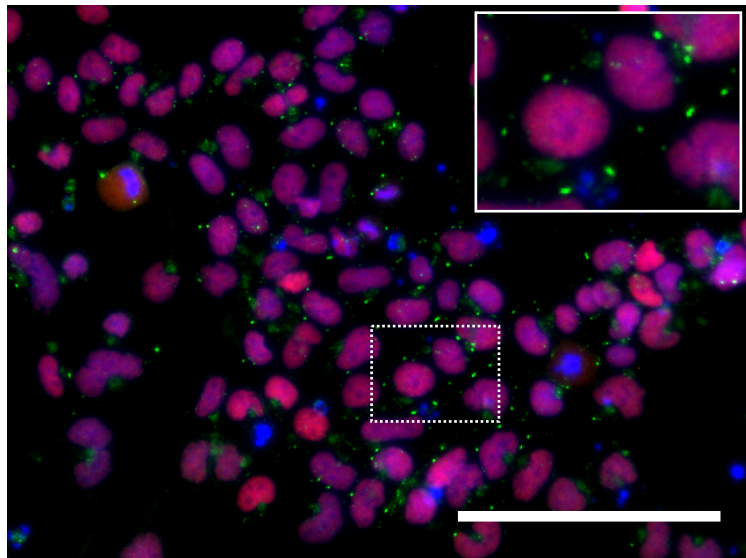

riPSC

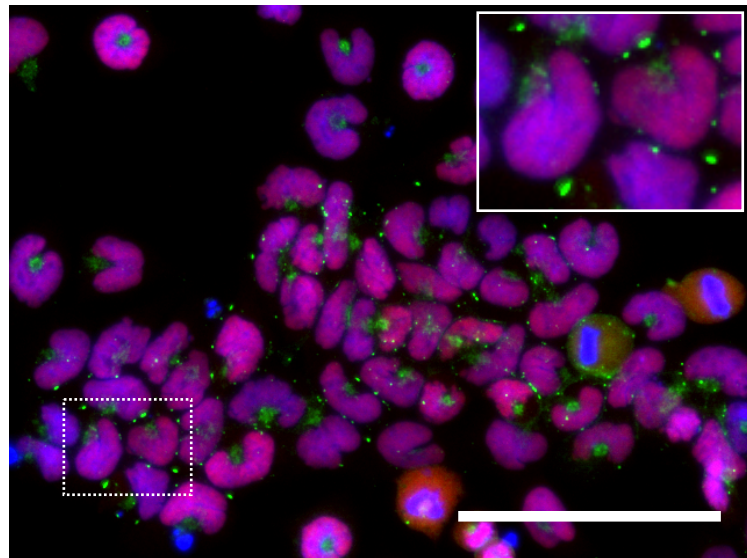

Supplement: S4 Fig — Expression of Connexin 43 protein (Cx43) was detected by immunofluorescence staining in both Oct4pos rPSC types. Scale bars: 100 μm. (PDF) [file pone.0192652.s004.pdf]

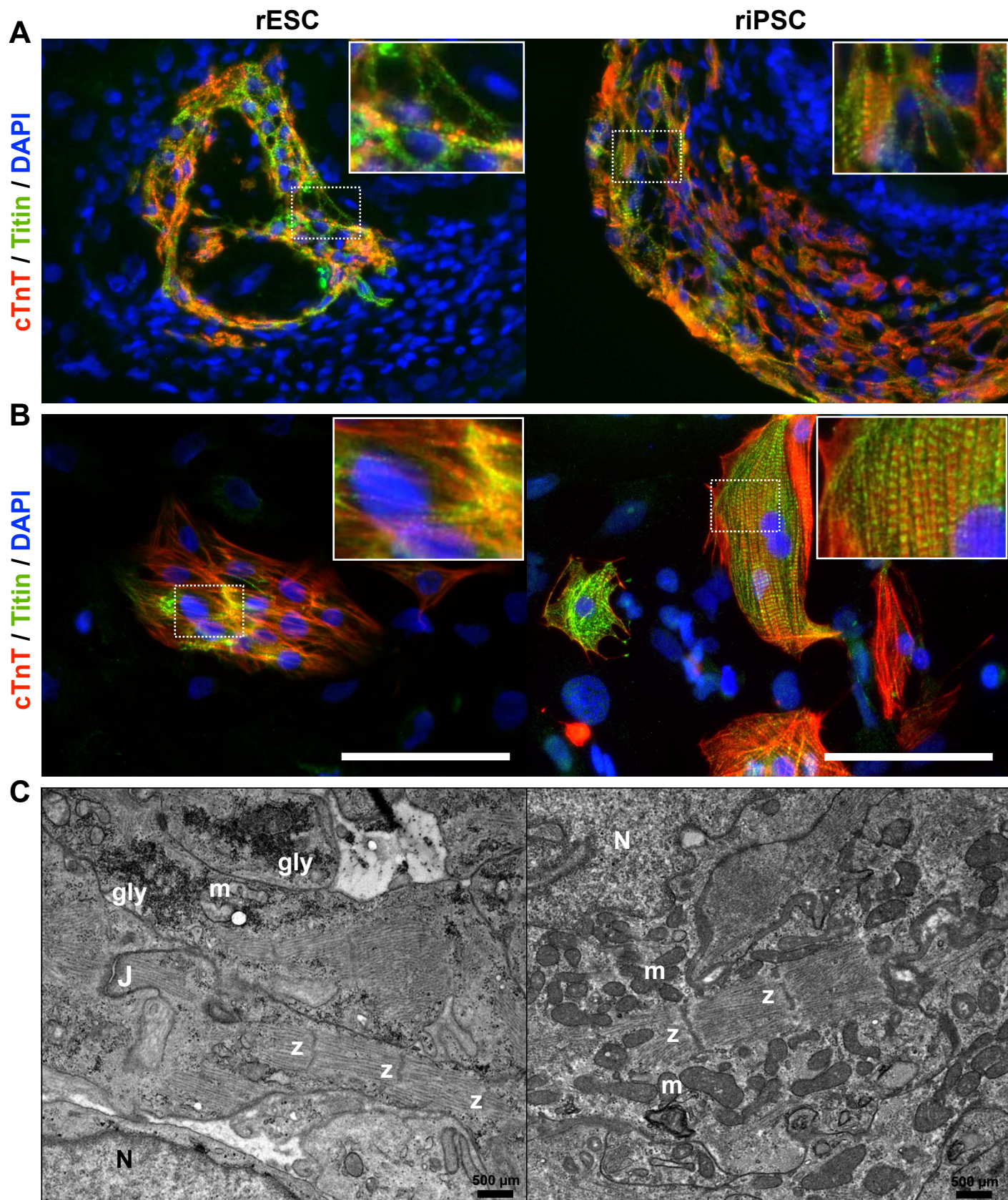

Supplement: S5 Fig — (A,B) Immunofluorescence stainings of EBs-cryosections of day 14 and plated cells for cardiac Troponin T and Titin. Nuclei are stained with DAPI. Scale bars: 100 μm. (C) Transmission electron microscopy images of EB sections. Z-bands (z), (m) mitochondria, (gly) glycogen, (N) nucleus, (J) intercellular junction. Scale bars: 500 nm. (PDF) [file pone.0192652.s005.pdf]

$\alpha$ -Actinin / Cx43 / DAPI

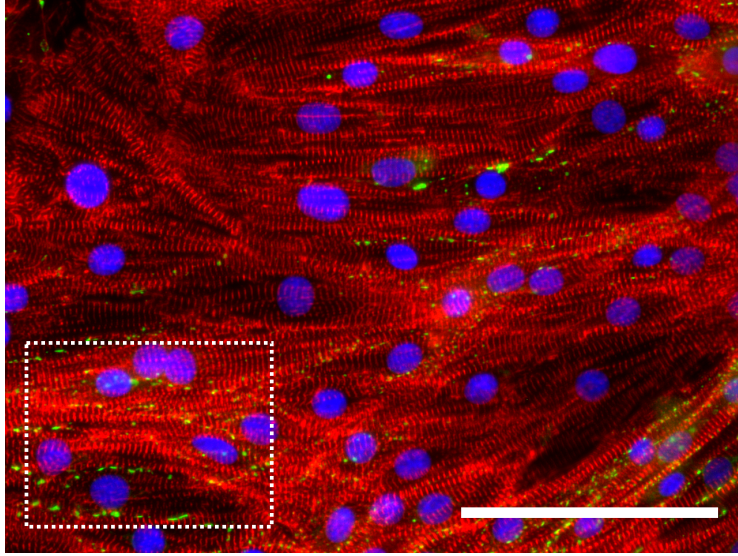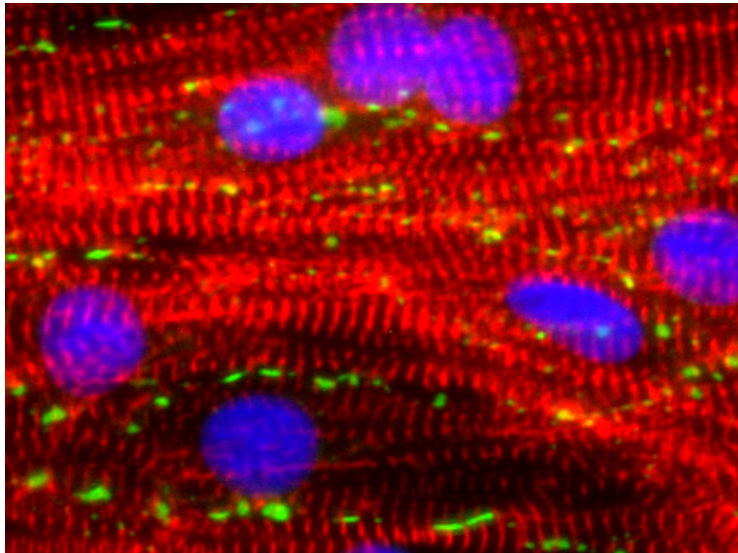

cTnT / Titin / DAPI

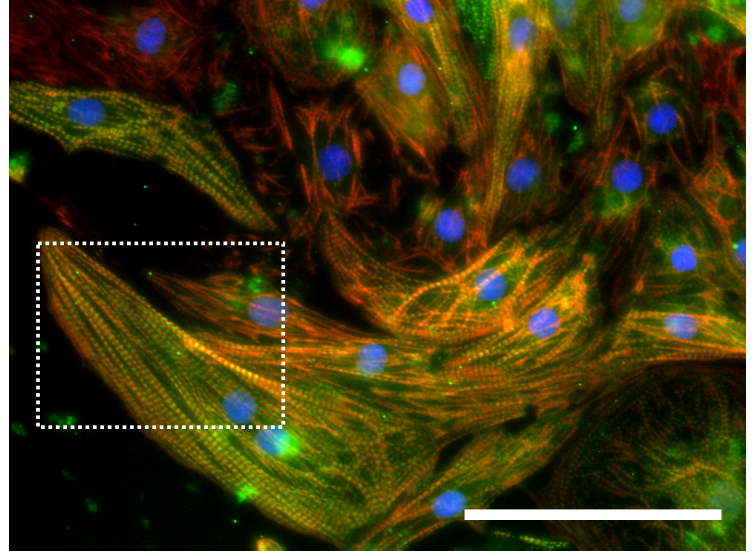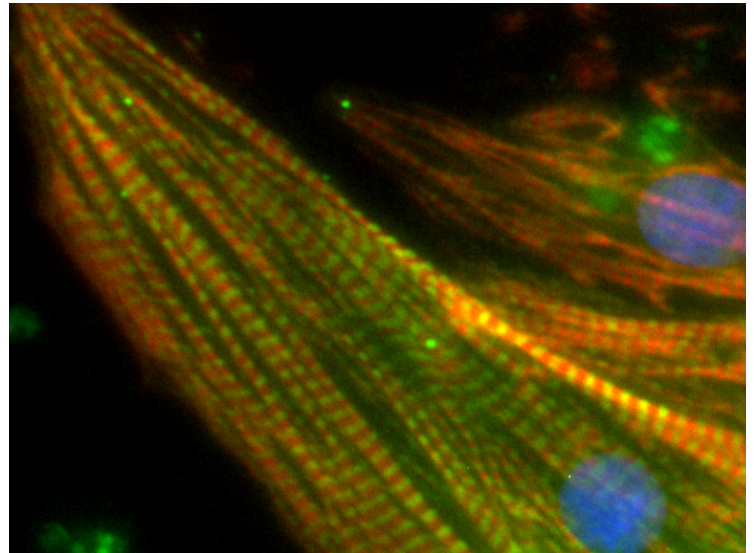

Supplement: S6 Fig — Scale bars: 100 μm. (PDF) [file pone.0192652.s006.pdf]
