# Supplementary material for: Generation of functional cardiomyocytes from rat embryonic and induced pluripotent stem cells using feeder-free expansion and differentiation in suspension culture
Source: PLoS One. 2018 Mar 7;13(3):e0192652. doi: 10.1371/journal.pone.0192652 (PMC5841662; doi:10.1371/journal.pone.0192652)
Supplement: S1 Table — (PDF) [file pone.0192652.s007.pdf]

S1 Table A: Primers and conditions for microsatellite genotyping.

Gene	Target	Position	Product (bp)	T_A	Cycles	5'->3' Sequence
**D1Rat122_for**	RGD ID	Chr 1	223 for Dark Agouti (rESC)	50	30	CTGCTCCACCTGCCTGTATT
**D1Rat122_rev**	38544		248 for Fischer344 (riPSC)			TCCCTTTGCAATAGACAATGG
**D2Rat250_for**	RGD ID	Chr 2	158 for Dark Agouti (rESC)	52	30	GTCCCTCTCCTGTCCCTCTC
**D2Rat250_rev**	41470		180 for Fischer344 (riPSC)			GAAGTCTGAACGCTCATGCA
**D3Rat17_for**	RGD ID	Chr 3	136 for Dark Agouti (rESC)	50	30	TCATTTTCCTTCCTCTCTCTCA
**D3Rat17_rev**	36717		174 for Fischer344 (riPSC)			AAGACAAAATGCTGGAGGGA

S1 Table B: Primers and conditions for semiquantitative RT-PCR.

Gene	Target	Position	Product (bp)	T_A	Cycles	5'->3' Sequence
**rRex1_for**	GI:1046864754	697	367	60	30	TTCTTGCCAGGTTCTGGAAGC
**rRex1_rev**		1063				TTTCCCACACTCTGCACACAC
**rNanog_for**	GI:672050892	1053	144	66	30	GCCACTAGGGAAAGCCAGGCG
**rNanog_rev**		1196				AAGAAAGCTGACCCTGGCCCTG
**rOct4_for**	GI:260269517	178	159	55	30	CCTGGCTAAGCTTCCAGGGGC
**rOct4_rev**		336				CCACGCCAACTTGGGGGACTA
**rGATA4_for**	GI:1046861721	778	146	62	33	GCTTCTGGAGCCACCTCGGG
**rGATA4_rev**		923				AAGGAGAAGCGCGGGGACAC
**rNkx2.5_for**	GI:16758951	383	159	58	34	GTGACCCTGACCCCGCCAAG
**rNkx2.5_rev**		541				AGACCTGCGCCTGCGAGAAG
**rα-MHC_for**	GI:1046861082	182	276	60	30	GATGCCCAGATGGCTGACTT
**rα-MHC_rev**		457				GGTCAGCATGGCCATGTCCT
**rß-MHC_for**	GI:672078038	5812	200	62	30	CGGAGGAACAGGCCAACACCA
**rß-MHC_rev**		6011				GTCTCAGGGCTTCACAGGCATCC
**rMlc2v_for**	GI:386869342	459	158	62	30	CCTGACGTCACCGGCAACCTT
**rMlc2v_rev**		616				TGGGATGGTGAACACCCGCAG
**rMlc2a_for**	GI:672076613	33	133	55	30	GGGCCTTTGGGTGATAAGGTTC
**rMlc2a_rev**		165				CACGTTGAGCCTGCTTGGTG
**rANP_for**	GI: 158341690	157	268	62	30	ATACAGTGCGGTGTCCAACA
**rANP_rev**		424				AGCCCTCAGTTTGCTTTTCA
**rCx40_for**	GI:9506724	876	171	54	30	TCCTGACTTCAACCAGTGCC
**rCx40_rev**		1046				GGCTTCTGGCCATACTGTGT
**rCx43_for**	GI:33285446	133	119	55	30	AGGAGTTCCACCAACTTTGGC
**rCx43_rev**		251				TGGAGTAGGCTTGGACCTTGTC
**rCx45_for**	GI:1046848581	1188	112	60	30	AAGAGCAGAGCCAACCAAAA
**rCx45_rev**		1299				CCCACCTCAAACACAGTCCT
**GAPDH_for**	GI:18042882	555	352	50	30	GGCCAAGGTCATCCATGA
**GAPDH_rev**		907				TCAGTGTAGCCCAGGATG

T_A: Annealing temperature
RGD ID: Rat Genome Database Identifier
