# Supplementary material for: Generation of functional cardiomyocytes from rat embryonic and induced pluripotent stem cells using feeder-free expansion and differentiation in suspension culture
Source: PLoS One. 2018 Mar 7;13(3):e0192652. doi: 10.1371/journal.pone.0192652 (PMC5841662; doi:10.1371/journal.pone.0192652)
Supplement: S2 Table — (PDF) [file pone.0192652.s008.pdf]

S2 Table: Test lots of fetal calf serum.

	Vendor	Order number	Lot number
FCS-1	Life Technologies	10270098	42G7651K
FCS-2	Sigma Aldrich	F7524	BCBQ7892V
FCS-3	Sigma Aldrich	F7524	BCBQ7890V
FCS-4	Sigma Aldrich	F7524	025M3355
FCS-5	Life Technologies	10270098	41F5635K
FCS-6	Life Technologies	10270098	41A1119K
