# Supplementary material for: Generation of functional cardiomyocytes from rat embryonic and induced pluripotent stem cells using feeder-free expansion and differentiation in suspension culture
Source: PLoS One. 2018 Mar 7;13(3):e0192652. doi: 10.1371/journal.pone.0192652 (PMC5841662; doi:10.1371/journal.pone.0192652)
Supplement: S3 Table — (PDF) [file pone.0192652.s009.pdf]

S3 Table A: Primary antibodies used for immunofluorescence stainings and flow cytometry

Species	Class	Name	Clonality	Vendor	Dilution
**Mouse**	IgM	Anti-Titin	Monoclonal	DSHB (Developmental Studies Hybridoma Bank)	1:30
**Mouse**	IgG1	Anti-sarcomeric-α-actinin	Monoclonal	Sigma-Aldrich	1:4000
**Mouse**	IgG1	Anti-cardiac troponin T	Monoclonal	Thermo Scientific	1:50
**Mouse**	IgM	Anti-Connexin 43	Monoclonal	Sigma-Aldrich	1:4000
**Mouse**	IgG2b	Anti-Oct 3/4	Monoclonal	Santa Cruz Biotechnology, INC	1:100

S3 Table B: Secondary antibodies used for immunofluorescence stainings and flow cytometry

Species	Name	Label	Vendor	Dilution
**Goat**	Anti-mouse IgM (μ)	Cy2	Jackson ImmunoResearch	1:200
**Goat**	Anti-mouse IgG (Fcγ, subclasses 1+2a+2b+3)	Alexa Fluor 488	Jackson ImmunoResearch	1:500
**Goat**	Anti-mouse IgG (Fcγ)	Cy3	Jackson ImmunoResearch	1:200
**Donkey**	Anti-mouse IgG (H+L)	Cy3	Jackson ImmunoResearch	1:200
